# Fluorogenic Coupled Assays Reveal Catalytic Properties, Inhibition Constants and Cellular Location of Mucin‐Active Carbohydrate Sulfatases

**DOI:** 10.1002/anie.2991471

**Published:** 2026-05-23

**Authors:** Charles W. E. Tomlinson, Madouc D. Bergers, David N. Bolam, Ana S. Luis, Alan Cartmell, Zachary Armstrong

**Affiliations:** ^1^ Department of Biology University of York York UK; ^2^ York Structural Biology Laboratory Department of Chemistry University of York York UK; ^3^ York Biomedical Research Institute University of York York UK; ^4^ Department of Bio‐organic synthesis Leiden Institute of Chemistry University of Leiden Leiden the Netherlands; ^5^ Biosciences Institute Faculty of Medical Sciences Medical School Newcastle University Newcastle upon Tyne UK; ^6^ Department of Medical Biochemistry and Cell Biology University of Gothenburg Gothenburg Sweden; ^7^ SciLifeLab University of Gothenburg Gothenburg Sweden

**Keywords:** biochemistry, carbohydrate, glycan, mucin, sialidase, sulfatase, sulfation

## Abstract

Sulfated glycans play a central role in human health and influence cell signaling, cancer progression, pathogen invasion, and host‐microbiome interactions. Metabolism of these glycans requires a specialized class of enzymes termed carbohydrate sulfatases. These enzymes are particularly important in the human gut where sulfated colonic mucin is produced and subsequently degraded by colonic bacteria. Despite the biological importance of carbohydrate sulfatases, there is currently a lack of chemical tools to study their activity, substrate selectivity, inhibition, and the discovery of novel enzymes. To address this, we have synthesized new chemical tools to rapidly and quantitatively determine the activity and selectivity of carbohydrate sulfatases in plate‐based coupled assays. We have synthesized 3‐*O*‐sulfated fluorogenic glycosides using efficient synthetic routes and combined these fluorogenic substrates with a glycosidase that selectively cleaves unsulfated glycosides, allowing sensitive detection of sulfatase activity on both purified protein and cell lysate from the S1_20 subfamily sulfatases. Furthermore, we show that the assay enables differentiation and quantification of substrate specificity, identification of sulfatase inhibitors, and determination of sulfatase (sub‐)cellular location for two S1_20 subfamily sulfatases. Collectively, we anticipate that these tools will further our understanding of the interplay between carbohydrate sulfatases, sulfated glycans, and human health.

## Graphical abstract

We report plate‐based coupled assays to rapidly and quantitatively determine the activity and selectivity of mucin‐active carbohydrate sulfatases. This fluorogenic assay further enables inhibitor identification and allows determination of (sub‐)cellular location.

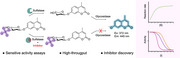

## 1 Introduction

Sulfated glycans are found at the surfaces of human cells, on proteoglycans in the extracellular matrix, and on secreted proteins, such as mucins present in the gut [1, 2, 3]. Turnover or degradation of these glycans requires a specialized class of enzymes termed carbohydrate sulfatases. These enzymes are particularly important in the gut where sulfated colonic mucin is produced by humans, and it is degraded by bacterial members of the colonic microbiota [4, 5]. This relationship is usually symbiotic, however, depletion of the colonic mucin by bacteria can lead to inflammatory bowel diseases and bacterial carbohydrate sulfatases have been implicated in these diseases [6, 7]. Furthermore, recent work has highlighted the importance of bacterial carbohydrate sulfatases for the colonic mucin‐degrading bacterium, *Bacteroides thetaiotaomicron* VPI‐5482 (*B. theta*), to utilize heavily sulfated colonic mucin [4, 5]. Notably, a single extracellular sulfatase (BT1636^3S‐Gal^ from the S1_20 subfamily) was essential to adequately metabolise sulfated mucin *O*‐glycans in vitro, and for competitive colonization in germ‐free mice [5].

**FIGURE 1:**
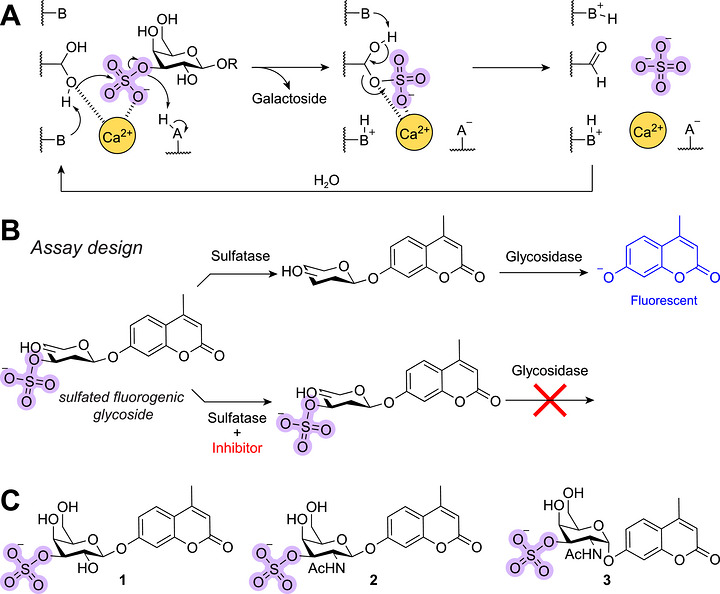
Sulfatase mechanism and coupled assay development. (A) S1 carbohydrate sulfatases employ an active site formylglycine to catalyse sulfate hydrolysis. The reaction mechanism is proposed to proceed through a transesterification‐elimination mechanism, that involves initial attack of the sulfate ester by the hydrated formylglycine. This leads to a covalent sulfated‐enzyme intermediate that can then undergo elimination, thereby releasing the sulfate and regenerating the formylglycine residue. This residue is then hydrated to restore the catalytic nucleophile. (B) Conceptual design of fluorogenic coupled assays to detect sulfatase activity. A sulfated carbohydrate bearing a fluorogenic group (here 4‐methylumbelliferone) linked at the anomeric position is incubated with a mixture of a carbohydrate sulfatase and a glycosidase. It is essential that the glycosidase can only cleave the desulfated glycoside, and not the sulfated glycoside. Fluorophore release directly reports the sulfatase hydrolysis rate, under conditions where the glycosidase is nonrate‐limiting. This assay can be used to detect both the presence of a carbohydrate sulfatase, and the decrease in activity due to the presence of an inhibitor. (C) Sulfated fluorogenic glycosides synthesized herein and employed in coupled assays.

Sulfatase sequences are classified into four families (S1–S4) and catalogued on the SulfAtlas database [8, 9]. Carbohydrate sulfatases are currently only found in the S1 family which comprises over 90% of all known sulfatase sequences. The S1 family is grouped into 110 subfamilies (denoted S1_X) based on sequence homology. This family of sulfatases employs a non‐genome encoded amino acid, formylglycine (FGly), generated from a Ser or Cys in the consensus sequence **C/S‐X‐P/A‐X‐R,** as their catalytic nucleophile and a His or Lys as their catalytic acid. S1 carbohydrate sulfatases are thought to hydrolyze sulfate esters via a transesterification‐elimination mechanism [10] (Figure 1A).

Several challenges are faced when characterizing S1 carbohydrate sulfatases. S1 sulfatases that act on arylsulfates, such as those acting on sulfated steroids [11], can be rapidly detected using aromatic, sulfated fluorogenic and chromogenic compounds [12]. However, this is not the case for many S1 carbohydrate sulfatases because these enzymes do not readily hydrolyze sulfated aromatics [13]. Alternative methods, such as detecting the pH change as sulfate groups are released, are time consuming, relatively insensitive, difficult to measure, and often low‐throughput [14]. Turbidity measurements using barium are insensitive and slow [15] and a capillary electrophoresis method linked to fluorescence detection, developed using a Perkin Elmer EZreaderII, is no longer commercially available and required expensive consumables [16]. Additionally, qualitative assays such as thin layer chromatography (TLC) and high‐performance anion exchange chromatography (HPAEC) appear to show incomplete substrate hydrolysis at the high concentrations of substrate used [4]. Coupled assays, employing a sulfated fluorogenic substrate and a glycosidase that can cleave the desulfated glycoside have previously been developed for select carbohydrate sulfatases [17, 18, 19, 20, 21, 22], however these methods have not yet been developed for S1_20 sulfatases. Thus, there is a need for fast, sensitive, facile, and high‐throughput technologies to assess S1_20 carbohydrate sulfatase activity.

We describe the development of a fluorescent assay for the rapid determination of carbohydrate sulfatase activity. This assay employs a sulfated carbohydrate (galactose or *N*‐acetylgalactosamine here) linked to a fluorogenic aglycone (Figure 1B). When the sulfated reporter is cleaved by the requisite sulfatase, a desulfated fluorogenic carbohydrate is released. This fluorogenic carbohydrate can then be cleaved by a complementary glycosidase that is unreactive toward the sulfated substrate. Here, we have used this coupled assay method to interrogate S1_20 sulfatases from the human symbiont *Bacteroides thetaiotaomicron*. We have synthesized 4‐methylumbelliferyl 3‐*O*‐sulfo‐β‐D‐galactopyranoside (MU‐β‐3S‐Gal, **1**), 4‐methylumbelliferyl 3‐*O*‐sulfo‐*N*‐acetyl‐β‐D‐galactopyranosaminide (MU‐β‐3S‐GalNAc, **2**) and 4‐methylumbelliferyl 3‐*O*‐sulfo‐*N*‐acetyl‐α‐D‐galactopyranosaminide (MU‐α‐3S‐GalNAc, **3**), see Figure 1C, as reporter molecules for S1_20 sulfatases. Using a coupled assay with β‐D‐galactosidase or *N*‐acetyl α‐/β‐D‐galactosaminidase, the synthesized substrates enable detection of S1_20 sulfatase activity in a 96‐well plate format via fluorescence readout. We use these substrates to determine Michaelis–Menten parameters for BT1636^3S‐Gal^ and its homologue BT1622^3S‐GalNAc/Gal^, demonstrating vastly different *K*_M_ values, as well as inhibition by different inorganic oxo‐anions and aluminum fluoride. We further use these substrates to demonstrate the cellular location of BT1636^3S‐Gal^ from cells in media. This demonstrates how sulfated, fluorescent glycosides, linked to an appropriate glycosidase, can be used to determine the biochemical properties and cellular location of carbohydrate sulfatases.

## 2 Results and Discussion

### 2.1 Expedient Synthesis of 4‐methylumbelliferyl‐3‐*O*‐sulfo‐glycosides

**SCHEME 1:**
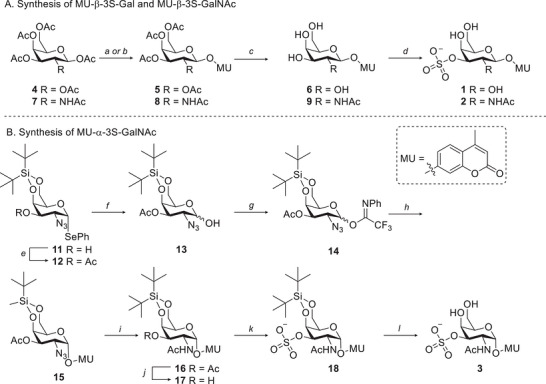
Synthesis of sulfated MU glycosides 1–3. *Reagents and conditions*: A. (a) *i*. 33 wt. % HBr in AcOH, DCM, 0°C; *ii*. tetrabutylammonium hydrogen sulfate, NaOH, 4‐methylumbelliferone, DCM/H_2_O (3:2 v/v), rt, (**5**: 79% over two steps); (b) *i*. 4 M HCl in dioxane, AcCl, 40°C; *ii*. tetrabutylammonium bromide, CsOH, 4‐methylumbelliferone, DCM/H_2_O (3:2 v/v), rt, (**8**: 35% over two steps); (c) NaOMe, MeOH, rt (**6**: 84%, **9**: 85%); (d) *i*. Bu_2_SnO, DMF/toluene (1:2 v/v), reflux; *ii*. SO_3_·Et_3_N, DMF, rt (**1**: 51% over two steps, **2**: 15% over two steps); B. (e) Ac_2_O, pyridine, 0°C to rt, (96%); (f) NIS, acetone/H_2_O (1:1 v/v), rt (88%); (g) 2,2,2‐trifluoro‐*N*‐phenylacetimidoyl chloride, Cs_2_CO_3_, acetone/water (50:1 v/v), 0°C → rt (97%); (h) 4‐methylumbelliferone, TMSOTf, DCM, −25°C to rt (67%); (i) *i*. H_2_, PtO_2_, THF, rt; *ii*. Ac_2_O, pyridine, 0°C → rt (85% over 2 steps); (j) NaOMe, MeOH, rt (86%); (k) SO_3_·pyridine, DMF, rt (90%); (l) 70% w/w HF‐pyridine, pyridine, 0°C to rt, (86%).

Although MU‐Gal is commercially available, we deemed this to be prohibitively expensive as a starting point for the synthesis of MU‐β‐3S‐Gal (**1**). We therefore commenced our synthetic route from the commercially available β‐D‐galactose pentaacetate (**4**) (Scheme 1A). Treatment of **4** with HBr in acetic acid furnished the corresponding α‐galactosyl bromide, which was coupled with 4‐methylumbelliferone under phase transfer conditions to afford peracetylated MU‐β‐galactoside **5** in a 79% yield over two steps. Zemplén deacetylation, followed by a regio‐selective 3‐*O*‐sulfation of the resulting unprotected MU‐Gal **6,** afforded MU‐3S‐Gal (**1**) [23, 24]. The regio‐selective sulfation was achieved by activating the 3‐OH through formation of a stannylene acetal bridging the cis‐diol, followed by treatment with SO_3_·Et_3_N. MU‐β‐3S‐GalNAc (**2**) was synthesized using a similar methodology (Scheme 1A). Peracetylated galactosamine **7** was converted into the corresponding anomeric chloride using HCl in AcCl, followed by a substitution with 4‐methylumbelliferone to afford fully protected β‐galactoside **8** (35% over two steps), according to literature precedent [25, 26]. Zemplén deacetylation, followed by a regio‐selective sulfation of unprotected MU GalNAc **9,** using the same stannylene acetal chemistry as used for its Gal equivalent **1**, furnished MU‐β‐3S‐GalNAc **2** ^23^.

For the introduction of the challenging α‐aryl glycosidic linkage in MU‐α‐3S‐GalNAc (**3**), we opted to use a di‐*tert*‐butylsilylidene (DTBS)‐functionalized donor (Scheme 1B). This glycosylation strategy, originally developed by Kiso and coworkers, is one of the most powerful methods to introduce 1,2‐cis linkages, and can even override neighboring group participation [27, 28]. Additionally, protection of the 3‐position with an orthogonal protecting group enables selective unmasking of this hydroxyl group for late‐stage sulfation. Therefore, the synthesis of MU‐α‐3S‐GalNAc (**3**) commenced with the acetylation of DTBS protected galactoside **10**, which was synthesized from commercially available tri‐*O*‐acetyl‐D‐galactal, using literature precedent [29, 30] (Scheme 1B). The anomeric selenoacetal of galactoside **12** was hydrolyzed using NIS in aqueous acetone to obtain hemiacetal **13** in 88% yield. Subsequently, trifluoroimidate **14** was synthesized by treatment of **13** with 2,2,2‐trifluoro‐*N‐*phenylacetimidoyl chloride and a mild base (97% yield) [31]. The resulting imidate donor **14** was immediately subjected to a TMSOTf‐promoted glycosylation to exclusively yield the α‐anomer **15** in a 67% yield. Azide **15** was reduced to the free amine using a Pt‐catalyzed hydrogenation, followed by acetylation using Ac_2_O to afford fully protected MU‐α‐GalNAc **16** in 85% over two steps. Deprotection of the acetyl group under Zémplen conditions resulted in alcohol **17** (86% yield), which was subjected to SO_3_·pyridine in DMF to afford 3S‐galactoside **18** (90% yield). Desilylation using HF‐pyridine resulted in MU‐α‐3S‐GalNAc (**3**) in an 86% yield.

### 2.2 S1_20 Enzymes Have Different Affinities for 3‐*O*‐sulfo‐D‐galacto‐configured Substrates

**FIGURE 2:**
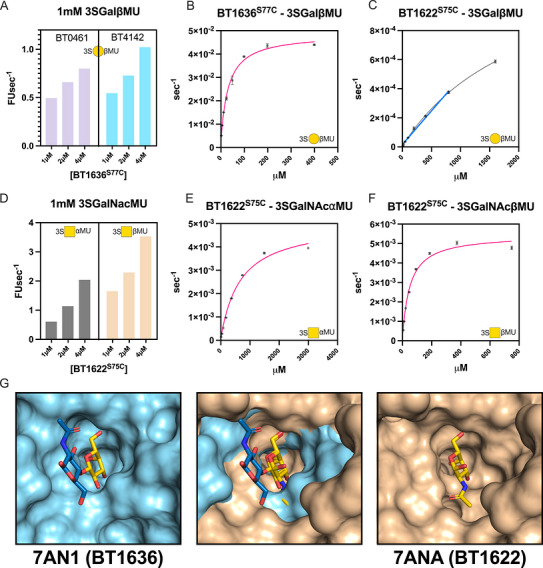
Using coupled assays to determine S1_20 carbohydrate sulfatase kinetics. A. Determination that BT1636^3S‐Gal^ dependent sulfate hydrolysis is the rate limiting step for the coupled reaction. B. Michaelis–Menten kinetics for BT1636^3S‐Gal^ with 4‐methylumbelliferyl 3‐*O*‐sulfo‐β‐d‐galactoside (**1**). C. Michaelis–Menten kinetics for BT1622^3S‐GalNAc/Gal^ with 4‐methylumbelliferyl 3‐*O*‐sulfo‐β‐D‐galactoside (**1**). D. Determination that BT1622^3S‐GalNAc/Gal^ dependent sulfate hydrolysis is the rate limiting step for the coupled reaction. E. Michaelis–Menten kinetics for BT1622^3S‐GalNAc/Gal^ using 4‐methylumbelliferyl 3‐O‐sulfo‐*N*‐acetyl‐α‐D‐galactoside (**3**). F. Michaelis–Menten kinetics for BT1622^3S‐GalNAc/Gal^ using 4‐methylumbelliferyl 3‐*O*‐sulfo‐*N*‐acetyl‐β‐D‐galactoside (**2**). All assays were carried out in triplicate in 10 mM HEPES pH 7.5 and 150 mM NaCl. Lines of best fit to hyperbolic equations are shown in pink, and a line of best fit to a linear equation is shown in blue. G. Surface representations of BT1636^3S‐Gal^ and BT1622^3S‐GalNAc/Gal^ in complex with their substrates, right and left respectively, with them overlaid in the middle panel.

Despite the proposed role of BT1636^3S‐Gal^ as a critical, high‐affinity sulfatase in *B. theta* for colonic mucin metabolism, no absolute measure of affinity or catalytic turnover of the recombinant enzyme has been reported [5]. Catalytic constants were determined using a coupled assay that employs a GH2 β‐galactosidase BT0461^GH2^, that cleaves 4‐methylumbelliferyl β‐d‐galactopyranoside (MU‐β‐Gal), at a sufficient concentration to render the sulfatases rate‐limiting (Figure 2A). Importantly, this galactosidase does not cleave MU‐β‐3S‐Gal (**1**). Using this assay, we determined the catalytic parameters, *K*_M_, *k*_cat_^app^, and *k*_cat_^app^/*K*_M,_ for BT1636^3S‐Gal^ and BT1622^3S‐GalNAc/Gal^ for the hydrolysis of MU‐β‐3S‐Gal (**1**). An apparent *k*_cat_ is reported as the amount of active enzyme is uncertain, due to incomplete conversion of the catalytic residue to a formyl glycine, and the values reported here were calculated using the total protein concentration. BT1636^3S‐Gal^ displayed a *K*_M_ of ∼30 µM and a *k*_cat_^app^ of 0.048 s^−1^, providing a *k*_cat_^app^/*K*_M_ of 1.61 × 10^3^ s^−1^ M^−1^ (Figure 2B and Table 1). In contrast, individual kinetic constants could not be established for BT1622^3S‐GalNAc/Gal^ using MU‐β‐3S‐Gal (**1**) as the reaction rates did not reach saturation at the highest substrate concentration. Only a *k*_cat_^app^/*K*_M_ of 4.62 × 10^−1^ s^−1^ M^−1^ could be established with a *K*_M_ of more than 1 mM (Figure 2C and Table 1).

**TABLE 1:** Michaelis constants determined for the hydrolysis of sulfated glycosides.

Protein	Substrate	*k*_cat_^app^ sec^−1^	*K*_M_ µM	*k*_cat_^app^/*K*_M_ sec^−1^M^−1^
BT1636^S77C^	MU‐β‐3S‐Gal	(4.9 ± 0.2) × 10^−2^	30 ± 4	(1.6 ± 0.2) x 10^3^
BT1622^S75C^	MU‐β‐3S‐Gal	—	—	(4.6 ± 0.1) x 10^−1^
BT1622^S75C^	MU‐α‐3S‐GalNAc	(5.0 ± 0.2) × 10^−3^	670 ± 80	(7.5 ± 0.9)
BT1622^S75C^	MU‐β‐3S‐GalNAc	(5.4 ± 0.2) × 10^−3^	50 ± 6	(1.1 ± 0.1) x 10^2^

The low activity of BT1622^3S‐GalNAc/Gal^ against MU‐β‐3S‐Gal (**1**) is consistent with its reported preference of 3‐*O*‐sulfated *N*‐acetylgalactosamine (3S‐GalNAc) [5]. To more accurately assess the kinetic properties of BT1622^3S‐GalNAc/Gal^, we repeated the experiment with MU‐β‐3S‐GalNAc (**2**) and MU‐α‐3S‐GalNAc (**3**). BT1622^3S‐GalNAc/Gal^ showed activity against both substrates but with a ∼14‐fold preference for MU‐β‐3S‐GalNAc (**2**) over MU‐α‐3S‐GalNAc (**3**), as determined from their respective *k*_cat_^app^/*K*_M_ (Figure 2 E,F). The *k*_cat_^app^ was similar for both substrates with the difference in activity mainly driven by the ∼12‐fold increase in *K*_M_ for the MU‐α‐3S‐GalNAc (**3**) substrate.

To detect the presence of produced MU‐β‐D‐GalNAc, we initially tested BVU_2198^GH123^, an *N*‐acetyl‐β‐d‐galactosaminidase [32], as a coupled enzyme. Unfortunately, BVU_2198^GH123^ had a small amount of activity on (**1**) (Figure S1). This necessitated the use of a different enzyme, BT4243^GH109^, that is active on both α‐ and β‐GalNAc and absolutely requires a free alcohol at the 3‐position for catalysis [33], to underpin the linked assay (Figure 2D). However, the activity from BVU_2198^GH123^ does highlight the possibility of using the chemical tools developed here, to identify glycosidases that are able to accommodate sulfated sugars. Indeed, recent studies have used a fluorogenic or chromogenic 6‐*O*‐sulfatated *N*‐acetylglucosaminide substrates to discover and characterize a 6‐sulfo‐*N*‐acetylglucosaminidases [34, 35]. Work by Loft et al. also used sulfated chromogenic β‐*N*‐acetylgalactosaminides and discovered fungal enzymes capable of cleaving the 3‐, 4‐, and 6‐*O*‐sulfated sugars [17].

The above data demonstrate that BT1636^3S‐Gal^ and BT1622^3S‐GalNAc/Gal^ have high and low affinities, respectively, for 3‐*O*‐sulfated D‐galactose. This is consistent with previous data, demonstrating that BT1636^3S‐Gal^ had a ∼10‐fold greater *k*_cat_^app^/*K*_M_ against BODIPY labelled 3‐*O*‐sulfated D‐galactose than BT1622^3S‐GalNAc/Gal^, with BT1622^3S‐GalNAc/Gal^ belonging to the subset of S1_20 sulfatases that are preferentially active on 3‐*O*‐sulfated‐*N*‐acetyl‐D‐galactosamine [5]. This preference for GalNAc displayed by BT1622^3S‐GalNAc/Gal^ was borne out using the MU‐β‐3S‐GalNAc (**2**) and MU‐α‐3S‐GalNAc (**3**) substrates. Although BT1622^3S‐GalNAc/Gal^ displayed a preference for MU‐β‐3S‐GalNAc (**2**), its ability to also cleave MU‐α‐3S‐GalNAc (**3**) at appreciable rates correlates with the more open active site surrounding its 0‐subsite, the region that accommodates the monosaccharide bearing the targeted sulfate, when compared to other sulfatases, such as BT1636^3S‐Gal^ (Figure 2G).

These data demonstrate that the aryl‐linked sulfo‐sugar substrates can be used to determine kinetic constants for S1_20 sulfatases targeting 3S‐Gal and 3S‐GalNAc and can differentiate between enzymes with high and low Michaelis constants; thereby providing a rapid biochemical tool for distinguishing between S1_20 members targeting 3‐*O*‐sulfated d‐galactose and 3‐*O*‐sulfated *N*‐acetyl‐d‐galactosamine.

### 2.3 Coupled Fluorogenic Sulfatase Assays Enable the Identification of Potent Inhibitors of S1_20 Sulfatases

Given that BT1636^3S‐Gal^ and its orthologues in other Bacteroidota species are potential drug targets, we envisioned that the coupled fluorogenic sulfatase assays developed here could also be used to both identify and quantify inhibitors of this enzyme class. As a proof of principle, we evaluated whether several oxoanions and AlF_3,_ known transition‐state mimics of sulfatases or phosphatases, could inactivate BT1636^3S‐Gal^ or BT1622^3S‐GalNAc/Gal^. We rationalized that these species could inhibit the above enzymes, as vanadate is a known inhibitor of S1_11 carbohydrate sulfatases [16] and oxoanions have previously been reported as inhibitors of an aryl sulfatase [36].

**FIGURE 3:**
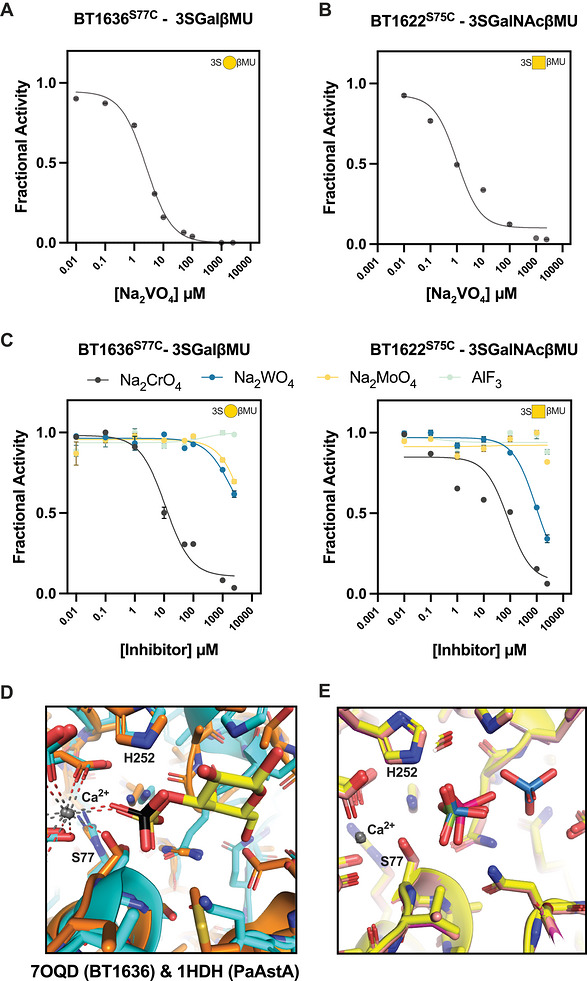
Orthovanadate is a potent inhibitor of S1_20 carbohydrate sulfatases. A. Dose‐response curve for inhibition of BT1636^3S‐Gal^ by orthovanadate using 1 mM **1** as substrate. B. Dose‐response curve for inhibition of BT1622^3S‐GalNAc/Gal^ by orthovanadate using 1 mM of **1** as substrate. C. Dose‐response curves for the inhibition of BT1636^3S‐Gal^ or BT1622^3S‐GalNAc/Gal^ by metal complexes using 1 mM **1** or **2** (respectively) as substrate. All assays were carried out in triplicate in 10 mM HEPES pH 7.5 and 150 mM NaCl. D. Stick representation of 1HDH (orange) and 7OQD (aqua) structures, showing substrate sulphate (HDH—black) and 3‐O‐sulfo‐β‐D‐galactopyranose (7OQD—yellow) orientations. E. Stick representation of crystals structures (determined here) of BT1636 soaked with phosphate (PDBID: **9THU;** two blue ions), chromate (PDBID: **9THV**; green ion), and molybdate (PDBID: **9THW**; pink ion) showing distinct inhibitor ion conformations.

Inhibition assays performed in the presence of the above species (Figure 3, Table 1) reveal that, indeed, vanadate can block the action of both BT1636^3S‐Gal^ and BT1622^3S‐GalNAc/Gal^ sulfatases with IC_50_ values of 2.6 ± 0.5 and 1.0 ± 0.5 µM, respectively. Furthermore, chromate and tungstate also showed inhibition against both enzymes, although to a lesser extent than vanadate. In contrast, molybdate only showed a small percentage of inhibition against BT1636^3S‐Gal^ at the highest concentrations (Figure 3D,E, Table 1). The trigonal planar compound AlF_3_, which has previously been shown to structurally mimic the transition‐state of phosphatases [37], did not appreciably inhibit either protein (Figure 3C,D,E, Table 1). Using these assays, we were also able to observe inhibition of BT1636^3S‐Gal^ by tungstate and vanadate, in whole cells at concentrations close to those described above, and without substantially affecting cell viability (Figure S2).

An additional benefit of being able to determine the Michaelis constant (*K*_M_), vide supra, with these coupled assays is that the *K*_M_ can be used, alongside the IC_50_, and substrate concentration to calculate inhibition rate constants via the Cheng–Prusoff equation [38], (Table 1). Inhibition constants (*K_I_*) of 0.34 ± 0.07 and 0.11 ± 0.02 µM were determined for orthovanadate inhibition of BT1636^3S‐Gal^ and BT1622^3S‐GalNAc/Gal^, respectively. This is ∼1000‐fold lower than the 117.6 µM *K*_I_ value of orthovanadate for the S1_11 sulfatase BT4656^6S‐GlcNAc/GlcNS^ that de‐sulfates *6‐O‐*sulfated *N*‐acetyl‐d‐glucosamine and 6‐*O*‐sulfated *N*‐sulfo‐d‐glucosamine [16]. This suggests that, despite high structural similarity of the sulfate binding site, differences in inhibitor affinities do exist, which could be exploited to develop tailored inhibitors involving tetrahedral metal complexes. These data demonstrate not only the utility of these fluorogenic substrates, but also the importance of using an appropriate substrate when determining IC_50_ values, which are significantly affected by substrate concentration and the enzyme specific Michaelis‐constant (*K*_M_) for said substrate.

To better understand how the above transition metal oxoanions inhibit S1_20 sulfatases, we determined crystal structures of BT1636^3S‐Gal^ in complex with chromate and molybdate, as well as inorganic phosphate (Figures 3 and S3 and Table 2). The three oxoanions all adopt product orientations with their tetrahedral configuration ‘inverted’ compared to that observed in the sulfated substrate (PDB: 7OQD), and free sulfate observed in the orthologue PaAsta (PDB: 1HDH). These complexes indicate that the oxoanion inhibitors chromate and molybdate mimic product complexes, thus working through a similar binding mode observed in product inhibition. Although we were unable to obtain a crystal structure of the bound ortho vanadate, we suspect the enhanced inhibition by ortho vanadate may be due to the metal oxoanion adopting a conformation more closely mimicking a substrate or through covalent inhibition.

**TABLE 2:** Inhibition constants determined for oxoanions and aluminum fluoride.

	BT1636^S77C –^ 3SGalβMU		BT1622^S75C –^ 3SGalNAcβMU	
Inhibitor	IC_50_ (µM), [**1**] = 200 µM	K_I_^calc^ (µM)	IC_50_ (µM), [**2**] = 400 µM	K_I_^calc^ (µM)
**VO_4_^2−^**	2.6 ± 0.5	0.34 ± 0.07	1.0 ± 0.5	0.11 ± 0.02
**CrO_4_^2−^**	11 ± 4	1.5 ± 0.5	80 ± 80	9 ± 9
**WO_4_^2−^**	2000 ± 1000	300 ± 200	1000 ± 400	100 ± 40
**MoO_4_^2−^**	>2500	NA	NA	NA
**AlF_3_**	NA	NA	NA	NA

### 2.4 Determining the Cellular Location of BT1636^3S‐Gal^ From Cells in Media

The cellular location of an enzyme can be as critical as its specificity in performing its biological function. Signal peptide prediction using computational tools, such as SignalP 6.0 [39] and psortb [40], are largely based on *Escherichia coli* annotations and the prediction of protein localisation in phyla such as Bacteroidota can be challenging. These tools can determine the presence of SPI or SPII signal peptides, with the latter resulting in a membrane anchored protein. This information alone is insufficient to definitively determine whether a protein is periplasmic or extracellular in Gram‐negative bacteria. Although SPI proteins are largely presumed to be periplasmic several reports have found them to be extracellular [41, 42], while proteins with an SPII signal peptide are membrane anchored [43] this anchoring can occur at the cell surface or in the periplasm. The gold standard for protein localization is to generate a specific antibody for the protein target and combine this with fluorescent microscopy, or protease protection assays and western blotting. Although these assays are specific, they are also expensive, time‐consuming, and antibody production can fail. *Bacteroides* species under aerobic conditions are metabolically inactive and the diffusion of the substrates developed here is limited and/or slow [44]. This implies that synthetic substrates such MU‐β‐3S‐Gal (**1**), and MU‐β‐3S‐GalNAc (**2**) could be used as a readout for cell surface activity.

**FIGURE 4:**
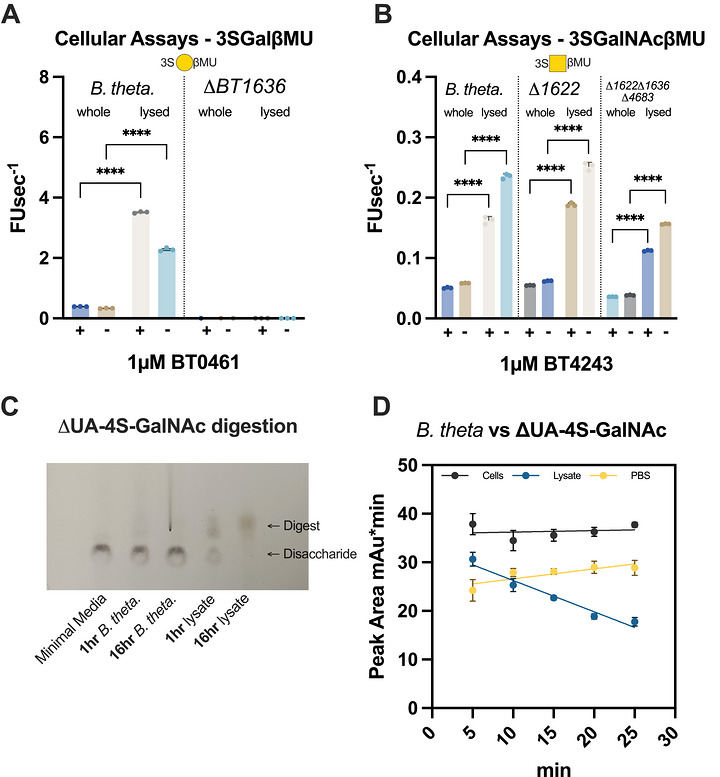
Determining sulfatase cellular location. A. Cell based assays conducted in minimal media using 200 µM 3‐*O*‐sulfated 4‐methylumbelliferone‐β‐d‐galactose with wildtype and Δ*bt1636* strains of *B. thetaiotaomicron*. B. Cell based assays conducted in minimal media using 200 µM 3‐*O‐*sulfated 4‐methylumbelliferyl‐β‐d‐*N*‐acetyl galactose with wildtype and Δ*bt1622*, Δ*bt1622/1636/4683* strains of *B. thetaiotaomicron*. C. Thin layer chromatography (TLC) analysis of PBS washed cells and lysates incubated with 5 mM of the disaccharide uronic acid β1,4 4‐*O*‐sulfated D‐GalNAc. D. High performance anion exchange chromatography of PBS washed cells and lysates incubated with 100 µM of the disaccharide α‐Δ4,5‐uronic acid‐(1→3)‐4‐*O*‐sulfo‐d‐GalNAc. All quantitative assays were conducted in triplicate whilst TLC analysis was performed in duplicate. In panels A and B significance of *p* < 0.0001 is denoted by ‘****’.

To investigate whether the above substrates could be used to determine subcellular enzyme location, we tested the ability of whole cells and sonicated lysates of wildtype *B. theta* and a Δ*bt1636* strain, grown on gastric mucin oligosaccharides (gMOs), to turnover MU‐β‐3S‐Gal (**1**) in the presence of BT0461^GH2^. Assays were conducted aerobically, in the cell growth media, using 200 µM of MU‐β‐3S‐Gal (1). Hydrolysis was detected for wildtype whole cells but not the Δ*bt1636* strain (Figure 4A). Sonicated lysates from wildtype cells also showed high activity, whilst the Δ*bt1636* strain showed no activity. These data are consistent with BT1636^3S‐Gal^ having both an extracellular and intracellular location. BT1636^3S‐Gal^ has previously been confirmed as having an extracellular location and although our data agrees with this conclusion, they also reveal that although ∼10% of BT1636^3S‐Gal^ is extracellular, 90% is intracellular (Figure 4A). BT1636^3S‐Gal^ has an SPI signal peptide thus the intracellular activity is deduced to be from enzyme located in the periplasm. The mechanism by which *B. theta* localises the SPI possessing BT1636^3S‐Gal^ to the cell surface is unknown. However, the GH33 sialidase, BT0455^GH33^, which also has an SPI signal peptide has also been reported to have both an extracellular and periplasmic location [45].

Although the use of a linked enzyme improved rates of glycoside hydrolysis, there was enough endogenous galactosidase activity both at the cell surface and from lysates that cleavage of MU‐Gal product occurred (Figure 4A). No activity was observed in the Δ*bt1636* strain demonstrating that the assay was reporting the activity of BT1636^3S‐Gal^ and not any other carbohydrate sulfatase, or a GH capable of hydrolysing MU‐β‐3S‐Gal (**1**) (Figure 4A).

Using the substrate MU‐β‐3S‐GalNAc (**2**) at 400 µM, and a similar method as above, we attempted to determine the cellular location of BT1622^3S‐GalNAc/Gal^. Similar activity was detected as observed for BT1636^3S‐Gal^, indicating the presence of activity at the cell surface but with greater activity observed from cell lysates (Figure 4B). This activity, however, was not lost when the cellular assays were repeated with the Δ*bt1622* strain, or the triple mutant Δ*bt1622/*Δ*bt1636/*Δ*bt4683* which is devoid of all 3S‐galacto‐targeting sulfatases (Figure 4B). This suggests there is either an unknown 3S‐GalNAc targeting sulfatase or a GH capable of hydrolysing MU‐β‐3S‐GalNAc (**2**). Identification of the enzyme responsible for this activity and whether it is a sulfatase from a different subfamily or a GH remains a question for future study.

As cross validation of our cell‐based strategy above, we confirmed the location of the GAG targeting sulfatase BT3349^4S‐UA‐4SGalNAc^, an enzyme known to be periplasmic [44]. Qualitative thin layer chromatography analysis, using 5 mM of the substrate Δ4,5‐uronic acid‐(1→3)‐4‐*O*‐sulfo‐d‐GalNAc (UA‐4SGalNAc), showed no hydrolysis of the substrate by whole cells after an incubation time of 1 and 16 h (Figure 4C). We then conducted the assay quantitatively over 25 min using high performance anion exchange chromatography with 100 µM UA‐4SGalNAc (Figure 4D). No reduction in substrate concentration is observed with whole cells or PBS controls while, cell lysates display a clear reduction in substrate concentration. These data confirm the cellular location of BT3349^Δ4,5UA‐4SGalNAc^ but critically show that diffusion of sulfated sugars, tested here, across the *B. theta* outer membrane is not observable under aerobic conditions; even at high substrate concentrations. It should be noted that these experiments used *B. theta* cells that were centrifuged and washed three times with PBS whilst the MU‐β‐3S‐Gal/GalNAc based assays were performed in minimal media. The cells underwent no mechanical or osmotic stress which whilst of no consequence for *B. theta* is certainly not the case for all colonic bacteria [46] adding expanded utility to the substrates and methodology deployed here. These data indicate that MU‐β‐3S‐Gal (**1**) can be used as a broad tool for investigating the cellular location of S1_20 sulfatases from Bacteroidota cells directly from the media, and potentially other colonic microbiota species.

## 3 Conclusions

The lack of rapid, scalable assays for carbohydrate sulfatases has hindered our understanding of this important class of enzymes. Here, we have demonstrated the utility of coupled assays employing fluorogenic sulfated carbohydrate substrates to determine the catalytic properties, inhibition constants, and cellular location of S1 carbohydrate sulfatases. These assays have allowed us to quantify the substrate specificity and localization of mucin active sulfatases of *B. theta*, a key member of the human gut microbiome. Furthermore, we show that, using these tools, we can identify inhibitors for the sulfatase BT1636^3S‐Gal^ that is essential for the degradation of sulfated mucins by *B. theta*. We have also used x‐ray crystal structures of these bound inhibitors to rationalize their potency. Together, these results establish the power of the tools developed here for exploring and understanding the action of carbohydrate sulfatases.

The assays disclosed here will be a starting point to develop techniques, including metagenomic enzyme discovery [47, 48, 49], directed evolution [50, 51], microbial phenotyping [52, 53] and in vivo imaging, that rely on robust and high‐throughput assays to enhance fundamental insight into sulfated carbohydrate metabolism. In addition, the synthesis of analogous chemical tools should facilitate the investigation of other sulfatases that currently lack sensitive or high‐throughput activity assays. This could be particularly interesting for the investigation of enzymes that desulfate glycosaminoglycans, glycolipids, N‐glycans and sulfated polysaccharides found in food, such as phorphyran [54, 55, 56]. We anticipate these tools will advance our understanding of carbohydrate sulfatases in host‐microbiome interactions and our search for molecules that can modulate these interactions.

## Author Contributions

**Charles W.E. Tomlinson**: writing – review and editing, investigation, methodology, visualization.**Madouc D. Bergers**: investigation, visualization, methodology, writing – review and editing. **David N. Bolam**: resources. **Ana S. Luis**: resources. **Alan Cartmell**: conceptualization, funding acquisition, writing – original draft, writing – review and editing, visualization, resources, supervision. **Zachary Armstrong**: conceptualization, funding acquisition, writing – original draft, writing – review and editing, visualization, resources, supervision.

## Conflicts of Interest

The authors declare no conflicts of interest.

## Supporting information




**Supporting File 1**: anie72868‐sup‐0001‐SuppMat.docx.

## Data Availability

The data that support the findings of this study are openly available in Protein Data Bank at https://www.rcsb.org/, reference number 9THU, 9THV, and 9THW.

## References

[1] J. D. Esko and U. Lindahl , “Molecular Diversity of Heparan Sulfate,” Journal of Clinical Investigation 108 (2001): 169–173, 10.1172/JCI13530. 11457867 PMC203033

[2] D. Soares da Costa , R. L. Reis , and I. Pashkuleva , “Sulfation of Glycosaminoglycans and Its Implications in Human Health and Disorders,” Annual Review of Biomedical Engineering 19 (2017): 1–26, 10.1146/annurev-bioeng-071516-044610. 28226217

[3] Y. Tobisawa , Y. Imai , M. Fukuda , and H. Kawashima , “Sulfation of Colonic Mucins by N‐Acetylglucosamine 6‐O‐Sulfotransferase‐2 and Its Protective Function in Experimental Colitis in Mice,” Journal of Biological Chemistry 285 (2010): 6750–6760, 10.1074/jbc.M109.067082. 20018871 PMC2825469

[4] A. S. Luis , A. Baslé , D. P. Byrne , et al., “Sulfated Glycan Recognition by Carbohydrate Sulfatases of the Human Gut Microbiota,” Nature Chemical Biology 18 (2022): 841–849, 10.1038/s41589-022-01039-x. 35710619 PMC7613211

[5] A. S. Luis , C. Jin , G. V. Pereira , et al., “A Single Sulfatase Is Required to Access Colonic Mucin by a Gut Bacterium,” Nature 598 (2021): 332–337, 10.1038/s41586-021-03967-5. 34616040 PMC9128668

[6] H. H. Tsai , A. D. Dwarakanath , C. A. Hart , J. D. Milton , and J. M. Rhodes , “Increased Faecal Mucin Sulphatase Activity in Ulcerative Colitis: A Potential Target for Treatment,” Gut 36 (1995): 570–576, 10.1136/gut.36.4.570. 7737566 PMC1382499

[7] C. Hickey , K. Kuhn , D. Donermeyer , et al., “Colitogenic Bacteroides Thetaiotaomicron Antigens Access Host Immune Cells in a Sulfatase‐Dependent Manner Via Outer Membrane Vesicles,” Cell host & microbe 17 (2015): 672–680, 10.1016/j.chom.2015.04.002. 25974305 PMC4432250

[8] T. Barbeyron , L. Brillet‐Guéguen , W. Carré , et al., “Matching the Diversity of Sulfated Biomolecules: Creation of a Classification Database for Sulfatases Reflecting Their Substrate Specificity,” PLoS ONE 11 (2016): e0164846, 10.1371/journal.pone.0164846. 27749924 PMC5066984

[9] M. Stam , P. Lelièvre , M. Hoebeke , E. Corre , T. Barbeyron , and G. Michel , “SulfAtlas, the Sulfatase Database: State of the Art and New Developments,” Nucleic Acids Research 51 (2023): D647–D653, 10.1093/nar/gkac977. 36318251 PMC9825549

[10] S. R. Hanson , M. D. Best , and C. H. Wong , “Sulfatases: Structure, Mechanism, Biological Activity, Inhibition, and Synthetic Utility,” Angewandte Chemie 43 (2004): 5736–5763, 10.1002/anie.200300632. 15493058

[11] S. M. Ervin , J. B. Simpson , M. E. Gibbs , et al., “Structural Insights into Endobiotic Reactivation by Human Gut Microbiome‐Encoded Sulfatases,” Biochemistry 59 (2020): 3939–3950, 10.1021/acs.biochem.0c00711. 32993284

[12] O. P. van Diggelen , et al., “A Fluorimetric Assay of Steroid Sulphatase in Leukocytes: Evidence for Two Genetically Different Enzymes With Arylsulphatase C Activity,” Journal of Inherited Metabolic Disease 12 (1988): 273–280, 10.1007/bf01799217. 2533306

[13] C. J. Crawford , C. W. E. Tomlinson , C. Gunawan , et al., “Arylsulfamates Inhibit Colonic Bacteroidota Growth through a Sulfatase‐independent Mechanism,” Proceedings of the National Academy of Sciences 122 (2025): e2414331122, 10.1073/pnas.2414331122. PMC12280919 40638084

[14] A. G. Hettle , C. Vickers , C. S. Robb , et al., “The Molecular Basis of Polysaccharide Sulfatase Activity and a Nomenclature for Catalytic Subsites in this Class of Enzyme,” Structure 26 (2018): p747–758e4, 10.1016/j.str.2018.03.012. 29681469

[15] J. Munoz‐Munoz , D. Ndeh , P. Fernandez‐Julia , G. Walton , B. Henrissat , and H. J. Gilbert , “Sulfation of Arabinogalactan Proteins Confers Privileged Nutrient Status to Bacteroides plebeius,” MBio 12 (2021): e0136821, 10.1128/mBio.01368-21. 34340552 PMC8406133

[16] D. P. Byrne , J. A. London , P. A. Eyers , E. A. Yates , and A. Cartmell , “Mobility Shift‐based Electrophoresis Coupled With Fluorescent Detection Enables Real‐time Enzyme Analysis of Carbohydrate Sulfatase Activity,” Biochemical Journal 478 (2021): 735–748, 10.1042/BCJ20200952. 33480417 PMC7897442

[17] K. J. Loft , P. Bojarová , K. Slámová , V. Křen , and S. J. Williams , “Synthesis of Sulfated Glucosaminides for Profiling Substrate Specificities of Sulfatases and Fungal β‐N‐Acetylhexosaminidases,” Chembiochem 10 (2009): 565–576, 10.1002/cbic.200800656. 19156788

[18] E. A. Karpova , Y. V. Voznyi , J. L. M. Keulemans , et al., “A Fluorimetric Enzyme Assay for the Diagnosis of sanfilippo Disease Type A (MPS IIIA),” Journal of Inherited Metabolic Disease 19 (1995): 278–285, 10.1007/bf01799255. 8803769

[19] W. He , Y. V. Voznyi , A. M. Boer , W. J. Kleijer , and O. P. van Diggelen , “A Fluorimetric Enzyme Assay for the Diagnosis of Sanfilippo Disease Type D (MPS IIID),” Journal of Inherited Metabolic Disease 16 (1993): 935–941, 10.1007/bf00711508. 8127069

[20] Y. V. Voznyi , J. L. M. Keulemans , and O. P. van Diggelen , “A Fluorimetric Enzyme Assay for the Diagnosis of MPS II (Hunter disease),” Journal of Inherited Metabolic Disease 24 (2001): 675–680, 10.1023/a:1012763026526. 11768586

[21] W. Fuchs , R. Navon , M. M. Kaback , and H. Kresse , “Tay‐Sachs Disease: One‐step Assay of β‐N‐acetylhexosaminidase in Serum With a Sulphated Chromogenic Substrate,” Clinica Chimica Acta 133 (1983): 253–261, 10.1016/0009-8981(83)90269-3. 6226458

[22] K. Clinch , G. B. Evans , R. H. Furneaux , et al., “Synthesis and Utility of Sulfated Chromogenic Carbohydrate Model Substrates for Measuring Activities of Mucin‐desulfating Enzymes,” Carbohydrate Research 337 (2002): 1095–1111, 10.1016/s0008-6215(02)00104-0. 12062525

[23] B. Guilbert , N. J. Davis , M. Pearce , R. T. Aplin , and S. L. Flitsch , “Dibutylstannylene Acetals: Useful Intermediates for the Regioselective Sulfation of Glycosides,” Tetrahedron: Asymmetry 5 (1994): 2163–2178, 10.1016/s0957-4166(00)86292-8.

[24] L. Wang , N. V. Pavlova , M. Yang , S. Li , Y. Li , and Y. C. Lee , “Synthesis of Aryl 3′‐sulfo‐β‐lactosides as Fluorogenic and Chromogenic Substrates for Ceramide Glycanases,” Carbohydrate Research 306 (1998): 341–348, 10.1016/S0008-6215(97)10082-9. 9648244

[25] H. Gold , S. Munneke , J. Dinkelaar , et al., “A Practical Synthesis of Capped 4‐methylumbelliferyl Hyaluronan Disaccharides and Tetrasaccharides as Potential Hyaluronidase Substrates,” Carbohydrate Research 346 (2011): 1467–1478, 10.1016/j.carres.2011.03.042. 21536258

[26] S. Park and I. Shin , “Profiling of Glycosidase Activities Using Coumarin‐Conjugated Glycoside Cocktails,” Organic Letters 9 (2007): 619–622, 10.1021/ol062889f. 17256948

[27] A. Imamura , H. Ando , H. Ishida , and M. Kiso , “Di‐tert‐butylsilylene‐Directed α‐Selective Synthesis of 4‐Methylumbelliferyl T‐Antigen,” Organic Letters 7 (2005): 4415–4418, 10.1021/ol051592z. 16178547

[28] A. Imamura , H. Ando , S. Korogi , et al., “Di‐tert‐butylsilylene (DTBS) Group‐directed α‐selective Galactosylation Unaffected by C‐2 Participating Functionalities,” Tetrahedron Letters 44 (2003): 6725–6728, 10.1016/s0040-4039(03)01647-2.

[29] D. van der Es , N. A. Groenia , D. Laverde , et al., “Synthesis of E. faecium Wall Teichoic Acid Fragments,” Bioorganic & Medicinal Chemistry 24 (2016): 3893–3907, 10.1016/j.bmc.2016.03.019. 26993744

[30] Y. V. Mironov , A. A. Sherman , and N. E. Nifantiev , “Homogeneous Azidophenylselenylation of Glycals Using TMSN3–Ph2Se2–PhI(OAc)2,” Tetrahedron Letters 45 (2004): 9107–9110, 10.1016/j.tetlet.2004.10.022.

[31] B. Yu and H. C. Tao , “Glycosyl Trifluoroacetimidates. Part 1: Preparation and Application as New Glycosyl Donors,” Tetrahedron Letters 42 (2001): 2405–2407, 10.1016/S0040-4039(01)00157-5.

[32] C. Roth , M. Petricevic , A. John , E. D. Goddard‐Borger , G. J. Davies , and S. J. Williams , “Structural and Mechanistic Insights into a Bacteroides Vulgatus Retaining N‐acetyl‐β‐galactosaminidase That Uses Neighbouring Group Participation,” Chemical Communications 52 (2016): 11096–11099, 10.1039/c6cc04649e. 27546776

[33] Q. P. Liu , G. Sulzenbacher , H. Yuan , et al., “Bacterial Glycosidases for the Production of Universal Red Blood Cells,” Nature Biotechnology 25 (2007): 454–464, 10.1038/nbt1298. 17401360

[34] R. K. Bains , S. A. Nasseri , F. Liu , J. F. Wardman , P. Rahfeld , and S. G. Withers , “Characterization of a New family of 6‐sulfo‐N‐acetylglucosaminidases,” Journal of Biological Chemistry 299 (2023): 105214, 10.1016/j.jbc.2023.105214. 37660924 PMC10570127

[35] M. Dong , Z. Chen , Y. He , R. Zallot , and Y. Jin , “Bioinformatics‐Facilitated Identification of Novel Bacterial Sulfoglycosidases That Hydrolyze 6‐Sulfo‐N‐acetylglucosamine,” ACS Bio & Med Chem Au 4 (2024): 342–352, 10.1021/acsbiomedchemau.4c00088. PMC11659886 39712202

[36] P. J. Stankiewicz and M. J. Gresser , “Inhibition of Phosphatase and Sulfatase by Transition‐state Analogs,” Biochemistry 27 (2002): 206–212, 10.1021/bi00401a031. 3280015

[37] M. Du , C. Lamoure , B. Muller , et al., “Artificial Evolution of an Enzyme Active Site: Structural Studies of Three Highly Active Mutants of Escherichia coli Alkaline Phosphatase,” Journal of Molecular Biology 316 (2002): 941–953, 10.1006/jmbi.2001.5384. 11884134

[38] C. Yung‐Chi and W. H. Prusoff , “Relationship between the Inhibition Constant (KI) and the Concentration of Inhibitor Which Causes 50 per Cent Inhibition (I50) of an Enzymatic Reaction,” Biochemical Pharmacology 22 (1973): 3099–3108, 10.1016/0006-2952(73)90196-2. 4202581

[39] H. Nielsen , K. D. Tsirigos , S. Brunak , and G. von Heijne , “A Brief History of Protein Sorting Prediction,” Protein Journal 38 (2019): 200–216, 10.1007/s10930-019-09838-3. 31119599 PMC6589146

[40] N. Y. Yu , J. R. Wagner , M. R. Laird , et al., “PSORTb 3.0: Improved Protein Subcellular Localization Prediction With Refined Localization Subcategories and Predictive Capabilities for all Prokaryotes,” Bioinformatics 26 (2010): 1608–1615, 10.1093/bioinformatics/btq249. 20472543 PMC2887053

[41] J. Briliūtė , P. A. Urbanowicz , A. S. Luis , et al., “Complex N‐glycan Breakdown by Gut Bacteroides Involves an Extensive Enzymatic Apparatus Encoded by Multiple co‐regulated Genetic Loci,” Nature Microbiology 4 (2019): 1571–1581, 10.1038/s41564-019-0466-x. PMC7617214 31160824

[42] The Role of Akkermansia Muciniphila Sulfatases in Colonic Mucin Utilisation, *BioRxiv*, 10.1101/2025.09.11.675649 (2025).

[43] F. Teufel , J. J. Almagro Armenteros , A. R. Johansen , et al., “SignalP 6.0 Predicts all Five Types of Signal Peptides Using Protein Language Models,” Nature Biotechnology 40 (2022): 1023–1025, 10.1038/s41587-021-01156-3. PMC9287161 34980915

[44] D. Ndeh , A. Baslé , H. Strahl , et al., “Metabolism of Multiple Glycosaminoglycans by Bacteroides Thetaiotaomicron Is Orchestrated by a Versatile Core Genetic Locus,” Nature Communications 11 (2020): 646, 10.1038/s41467-020-14509-4. PMC6994673 32005816

[45] J. Briliūtė , P. A. Urbanowicz , A. S. Luis , et al., “Complex N‐glycan Breakdown by Gut Bacteroides Involves an Extensive Enzymatic Apparatus Encoded by Multiple co‐regulated Genetic Loci,” Nature microbiology 4 (2019): 1571–1581, 10.1038/s41564-019-0466-x. PMC7617214 31160824

[46] The Role of Akkermansia Muciniphila Sulfatases in Colonic Mucin Utilisation. *BioRxiv*, 10.1101/2025.09.11.675649 (2025).

[47] Z. Armstrong , P. Rahfeld , and S. G. Withers , “Chemical Glycobiology Part A. Synthesis, Manipulation and Applications of Glycans,” Methods in Enzymology 597 (2017): 3–23. 28935108 10.1016/bs.mie.2017.06.001

[48] M. Taupp , K. Mewis , and S. J. Hallam , “The Art and Design of Functional Metagenomic Screens,” Current Opinion in Biotechnology 22 (2011): 465–472, 10.1016/j.copbio.2011.02.010. 21440432

[49] Z. Armstrong , K. Mewis , F. Liu , et al., “Metagenomics Reveals Functional Synergy and Novel Polysaccharide Utilization Loci in the Castor Canadensis Fecal Microbiome,” The ISME Journal 12 (2018): 2757–2769, 10.1038/s41396-018-0215-9. 30013164 PMC6193987

[50] J. F. Wardman , F. Liu , S. Vainauskas , et al., “Reshaping of a Glycoside Hydrolase Active Site through Expression‐Compensated Droplet‐Based Microfluidic Screening Provides Useful Tools for Glycomics,” ACS Central Science 11 (2025): 1993–2005, 10.1021/acscentsci.5c01227. 41142332 PMC12550630

[51] M. Gantz , S. Neun , E. J. Medcalf , L. D. van Vliet , and F. Hollfelder , “Ultrahigh‐Throughput Enzyme Engineering and Discovery in in Vitro Compartments,” Chemical Reviews 123 (2023): 5571–5611, 10.1021/acs.chemrev.2c00910. 37126602 PMC10176489

[52] L. Chen , L. J. Keller , E. Cordasco , M. Bogyo , and C. S. Lentz , “Fluorescent Triazole Urea Activity‐Based Probes for the Single‐Cell Phenotypic Characterization of Staphylococcus aureus,” Angewandte Chemie International Edition 58 (2019): 5643–5647, 10.1002/anie.201900511. 30768830 PMC6456404

[53] K. E. Beatty , M. Williams , B. L. Carlson , et al., “Sulfatase‐activated Fluorophores for Rapid Discrimination of Mycobacterial Species and Strains,” Proceedings of the National Academy of Sciences 110 (2013): 12911–12916, 10.1073/pnas.1222041110. PMC3740907 23878250

[54] J. Jung , E. N. Schmidt , H. Chang , et al., “Understanding the Glycosylation Pathways Involved in the Biosynthesis of the Sulfated Glycan Ligands for Siglecs,” ACS Chemical Biology 20 (2025): 386–400, 10.1021/acschembio.4c00677.39836965

[55] K. Honke , Y. Zhang , X. Cheng , N. Kotani , and N. Taniguchi , “Biological Roles of Sulfoglycolipids and Pathophysiology of Their Deficiency,” Glycoconjugate Journal 21 (2004): 59–62, 10.1023/B:GLYC.0000043749.06556.3d. 15467400

[56] J. Hehemann , A. G. Kelly , N. A. Pudlo , E. C. Martens , and A. B. Boraston , “Bacteria of the human Gut Microbiome Catabolize Red Seaweed Glycans With Carbohydrate‐active Enzyme Updates From Extrinsic Microbes,” Proceedings of the National Academy of Sciences 109 (2012): 19786–19791, 10.1073/pnas.1211002109. PMC3511707 23150581

