# Supplementary material for: Fluorogenic Coupled Assays Reveal Catalytic Properties, Inhibition Constants and Cellular Location of Mucin‐Active Carbohydrate Sulfatases
Source: Angew Chem Int Ed Engl. 2026 May 23;65(30):e2991471. doi: 10.1002/anie.2991471 (PMC13383068; doi:10.1002/anie.2991471)
Supplement: Supplementary file 1 — Supporting File 1: anie72868‐sup‐0001‐SuppMat.docx. [file ANIE-65-e2991471-s001.docx]

**Supporting Information**

**for**

**Fluorogenic coupled assays reveal catalytic properties, inhibition constants and cellular location of mucin-active carbohydrate sulfatases**

Authors: Charles W.E. Tomlinson^a,b,c^,*, Madouc D. Bergers^d^,*, David N. Bolam^e^, Ana S. Luis^f,g^, Alan Cartmell^a,b,c,†^, Zachary Armstrong^d,†^

^a^ : Department of Biology, University of York, York YO10 5DD, United Kingdom

^b^ : York Structural Biology Laboratory, Department of Chemistry, University of York, York YO10 5DD, United Kingdom

^c^ : York Biomedical Research Institute, University of York, York YO10 5DD, United Kingdom

^d^ : Department of Bio-organic synthesis, Leiden institute of Chemistry, University of Leiden, Einsteinweg 55, 2333 CC Leiden, The Netherlands

^e^ :Biosciences Institute, Faculty of Medical Sciences, Newcastle University,

Medical School, Newcastle upon Tyne NE2 4HH, United Kingdom

^f^ :Department of Medical Biochemistry and Cell Biology, University of Gothenburg, Gothenburg 405 30, Sweden

^g^ :SciLifeLab, University of Gothenburg, 41390 Gothenburg, Sweden

**Table of Contents**

[Supplemental Tables 2](#_Toc224132226)

[Supplemental Figures 4](#_Toc224132227)

[Biochemical Methods 7](#_Toc224132228)

[Chemical Synthesis 9](#_Toc224132229)

[General experimental procedures 9](#_Toc224132230)

[Synthesis of MU-β-3S-Gal 9](#_Toc224132231)

[Synthesis of MU-β-3S-GalNAc 10](#_Toc224132232)

[Synthesis of MU-α-3S-GalNAc 12](#_Toc224132233)

[References 17](#_Toc224132234)

[NMR spectra 18](#_Toc224132235)

# Supplemental Tables

**Table S1. Minimal media for *Bacteroides thetaiotaomicron* growth experiments**

| **Stock** | **100 ml** | |
| --- | --- | --- |
| (NH_4_)_2_SO_4_ | 0.1 | g |
| Na_2_CO_3_ | 0.1 | g |
| cysteine, free base | 0.05 | g |
| 1 M KPO_4_ pH 7.2 | 10 | ml |
| Vitamin K solution, 1mg/ml | 0.1 | ml |
| FeSO_4_, 0.4 mg/ml | 1 | ml |
| resazurin, 0.25 mg/ml | 0.4 | ml |
| Vitamin B_12_, 0.01 mg/ml | 0.05 | ml |
| Mineral Salts for defined medium | 5 | ml |
| 1.2 mg/ml Haematin in 0.2 M histidine pH 8.0 | 0.1 | ml |
| dH_2_O | 85 | ml |
|  | | |
| Mineral salts for defined media: | | |
|  | | |
| NaCl 18 g | | |
| CaCl_2_ 2H_2_O 0.53 g | | |
| MgCl_2_ 6H_2_O 0.40 g | | |
| MnCl_2_ 4H_2_O 0.20 g | | |
| CoCl_2_ 6H_2_O 0.20g | | |
|  | | |
| Dissolve in 1 litre and filter; 0.2 micron. | | |

Table S2. X-ray data collection and refinement statistics

| PDB ID | 9THU | 9THV | 9THW |
| --- | --- | --- | --- |
| Ligand - ID | Phosphate - PO4 | Chromate - CQ4 | Molybdate - MOO |
| Spacegroup | P2_1_2_1_2_1_ | P2_1_2_1_2_1_ | P2_1_2_1_2_1_ |
| Cell dimensions  a, b, c (Å)  α,β,γ (°) | 74.490, 88.140, 103.390 | 74.651, 87.594, 103.322 | 74.700, 87.260, 103.130 |
|  | 90.000, 90.000, 90.000 | 90.000, 90.000, 90.000 | 90.000 90.000 90.000 |
| Beamline | DLS - I04 | DLS - I04 | DLS - I04 |
| Wavelength | 0.691 | 0.9537 | 0.7601 |
|  | **Data processing - Overall (Outer Shell)** | | |
| Low resolution limit | 60.44 (1.42) | 66.81 (1.73) | 51.56 (1.42) |
| High resolution limit | 1.4 (1.4) | 1.7 (1.7) | 1.4 (1.4) |
| Rmerge(within I+/I-)* | 0.087 (1.997) | 0.108 (2.081) | 0.105 (3.83) |
| Rmerge(all I+ and I-)* | 0.091 (2.145) | 0.11 (2.152) | 0.11 (4.273) |
| Rmeas (within I+/I-)* | 0.094 (2.167) | 0.116 (2.242) | 0.114 (4.144) |
| Rmeas (all I+ & I-)* | 0.094 (2.233) | 0.115 (2.233) | 0.114 (4.442) |
| Rpim (within I+/I-) | 0.035 (0.838) | 0.044 (0.832) | 0.042 (1.575) |
| Rpim (all I+ & I-) | 0.025 (0.617) | 0.031 (0.593) | 0.031 (1.206) |
| Rmerge in top intensity bin* | 0.038 () | 0.042 () | 0.039 () |
| Number of observations | 1872076 (85305) | 1023234 (55274) | 1816506 (86861) |
| Number unique | 134250 (6570) | 75194 (3917) | 132964 (6486) |
| Mean((I)/sd(I)) | 14.9 (1.2) | 12.1 (0.4) | 12.1 (0.7) |
| Half-set correlation CC(1/2) | 0.999 (0.609) | 0.999 (0.658) | 0.999 (0.501) |
| Completeness % | 100 (100) | 100 (100) | 100 (100) |
| Multiplicity | 13.9 (13) | 13.6 (14.1) | 13.7 (13.4) |
| Filtered Mean(chi^2) | 1.03 (0.91) | 0.42 (0.08) | 1.03 (0.9) |
| Anomalous completeness % | 100 (99.9) | 100 (100) | 100 (100) |
| Anomalous multiplicity | 7.2 (6.6) | 7.1 (7.2) | 7.1 (6.8) |
| DelAnom CC(1/2) | 0.06 (0.004) | -0.184 (0.01) | 0.066 (0.023) |
| Mid-Slope of Anom Probability | 1.059 () | 0.39 () | 1.063 () |
|  | **Refinement Statistics** | | |
| R(Rfree) | 0.12 (0.14) | 0.15 (0.19) | 10.13 (0.16) |
| RMSD Bonds (Å) | 0.02 | 0.008 | 0.01 |
| RMDS Angles (°) | 1.9 | 1.7 | 1.8 |
| Mean B Factor | 18.91 (21.31) | 29.95 (32.54) | 21.03 (23.67) |
| Main Chain (Side Chain) |  |  |  |
| Ramachandran Outliers | 0.42% | 0.83% | 0.41% |

# Supplemental Figures


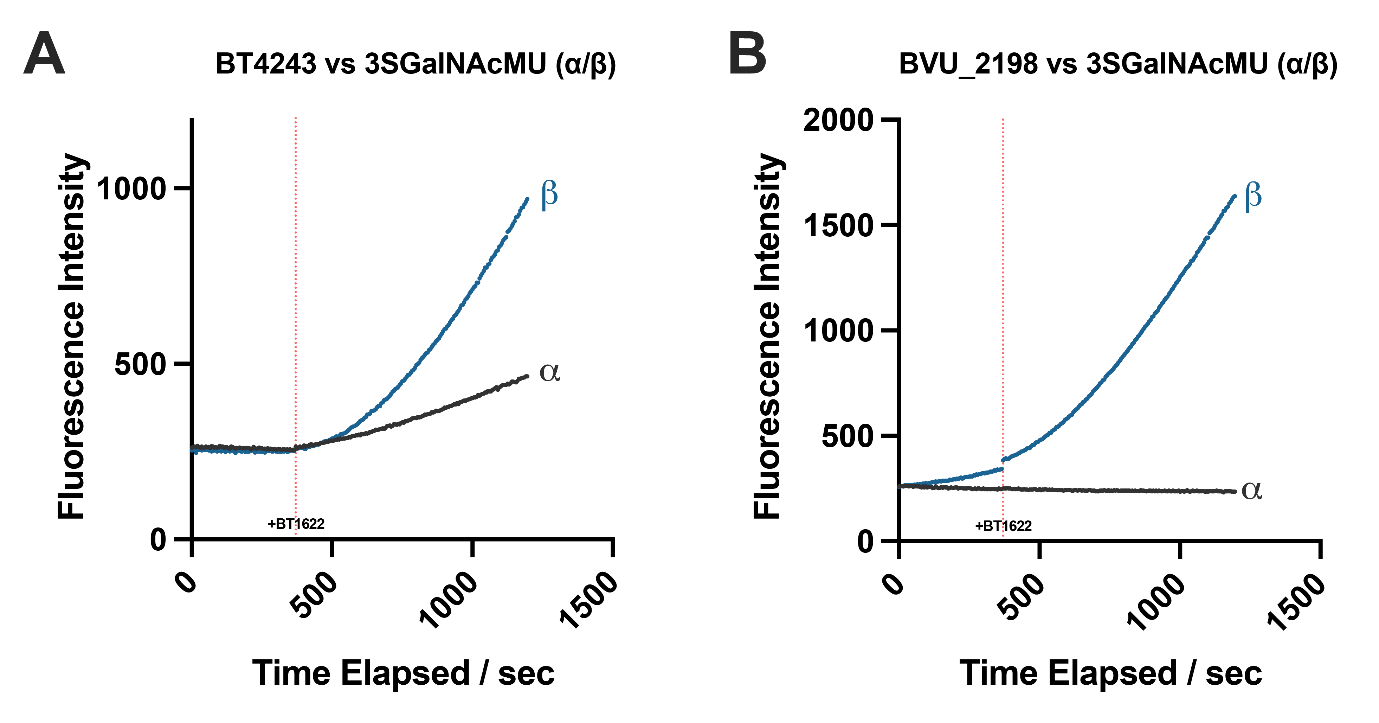


**Figure S1 Testing of glycosidases on 3-sulfated galactosaminides. A**. Activity of glycosidase BT4242 on MU-3S-GalNAc substrates (α/β) before, and after, addition of sulfatase BT1622. **B**. Activity of glycosidase BVU_2198 on MU-3S-GalNAc substrates (α/β) before, and after, addition of sulfatase BT1622. Clear activity on MU- β-3S-GalNAc prior to sulfate cleavage precludes BV0123 from use in a coupled assay.


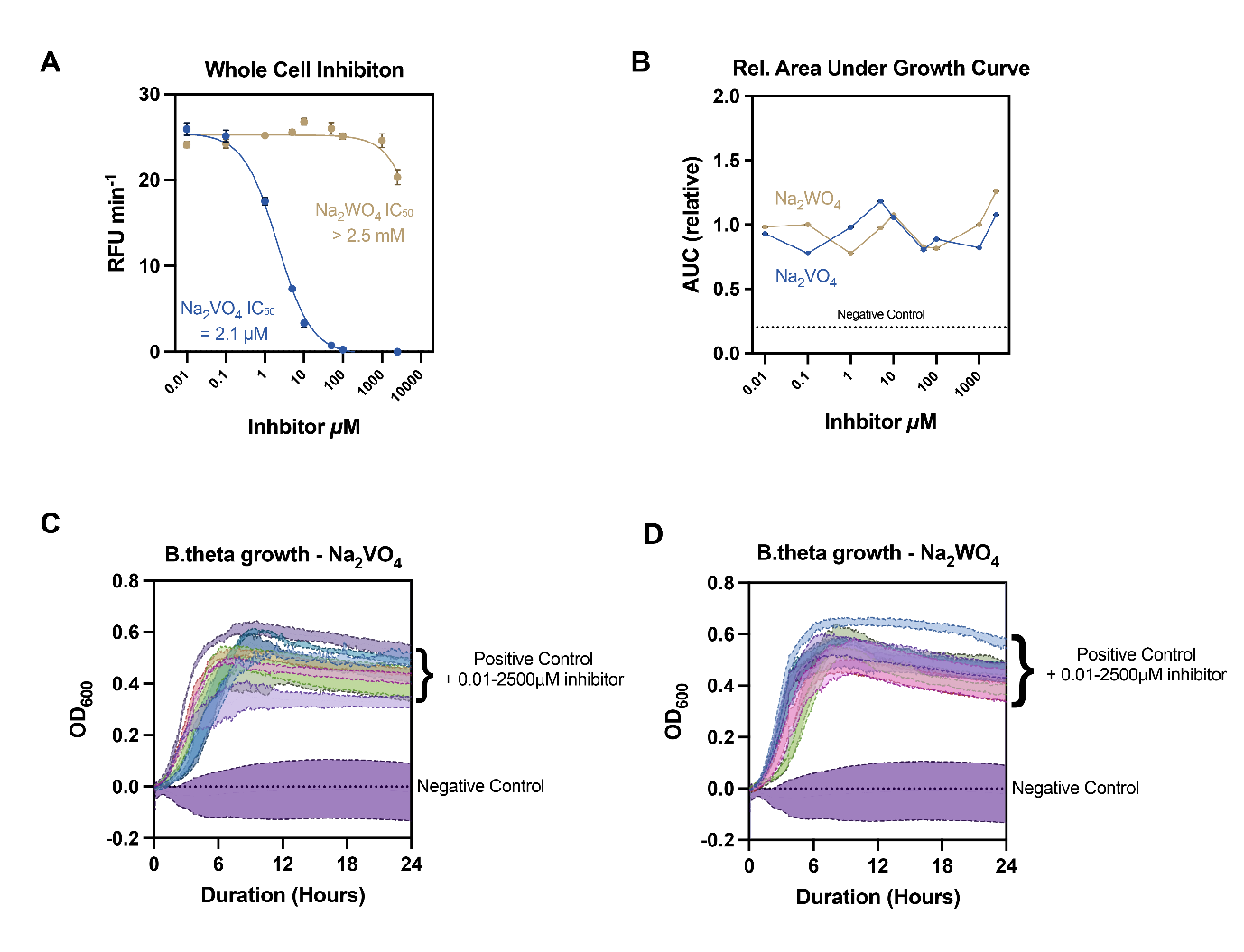


**Figure S2** Inhibition of BT1636 in whole cells. **A**. Inhibition curves for BT1636^3S-Gal^ performed in whole cells. **B**. Area under the curve for both growth of *B. theta* in the presence of sodium tungstate and sodium vanadate. **C**. Growth data for *B.theta* on rich media in the presence of 0.01-2500 uM sodium vanadate. **D.** Growth data for *B.theta* on rich media in the presence of 0.01-2500 uM sodium tungstate.

**
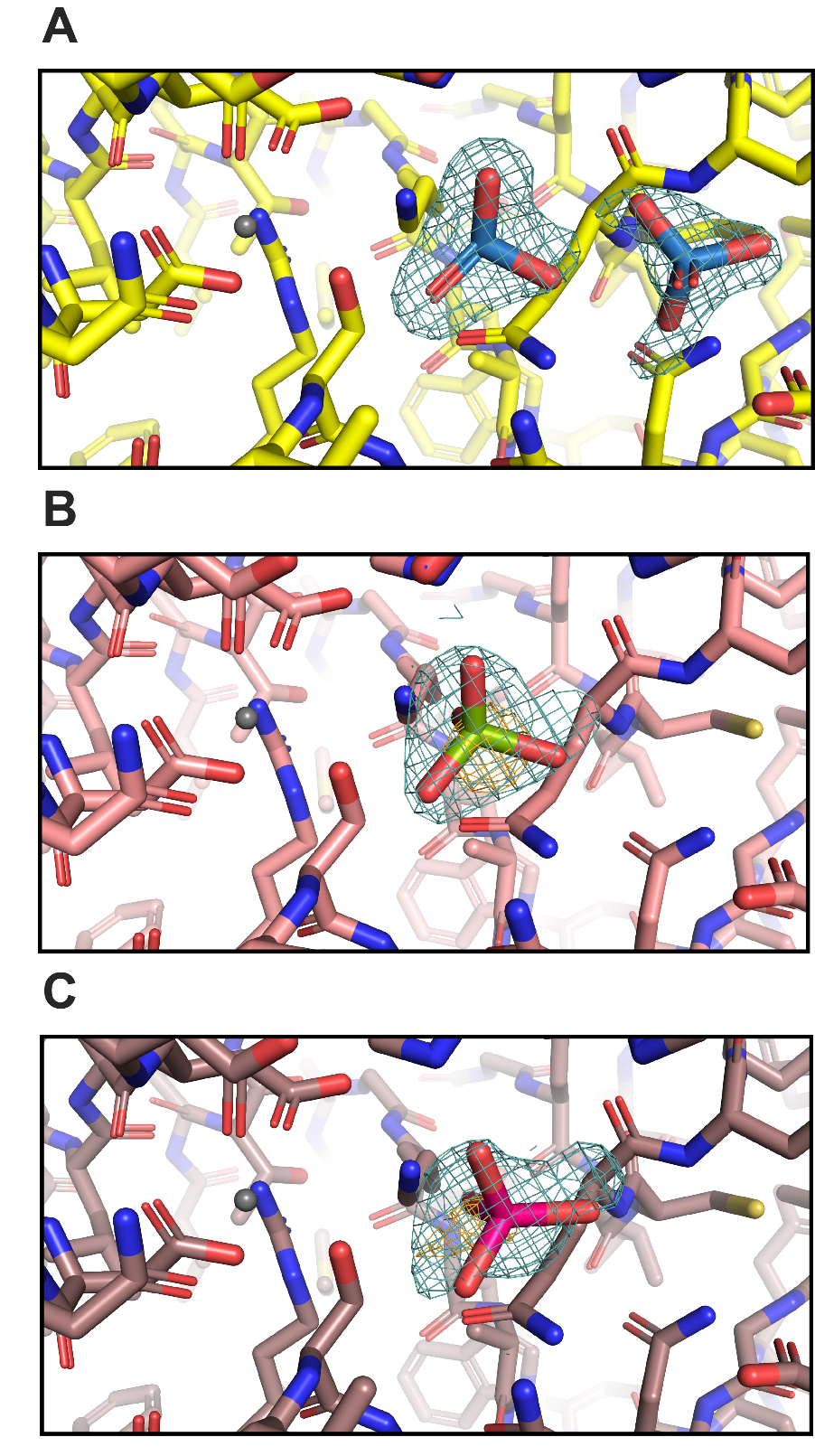
**

**Figure S3. 2F_o_-F_c_ and anomalous density maps. A.** 2F_o_-F_c_ density (teal mesh) for PBD 9THU with bound phosphate ions (blue). **B.** 2F_o_-F_c_ density (teal mesh) and anomalous density (orange mesh) for PDB 9THV with bound chromate ion (green). **C.** 2F_o_-F_c_ density (teal mesh) and anomalous density (orange mesh) for PDB 9THW with bound molybdate ion (pink).

# Biochemical Methods

**Recombinant enzyme expression and purification**

Sulfatase genes were amplified by PCR using the appropriate primers and the amplified DNA cloned in pET28b with N-terminal His_6_ tags using NheI/XhoI restriction sites. Recombinant sulfatase genes in pET28b were co-transformed with pBAD/myc-his A Rv0712 (a formylglycine generating enzyme from *Mycobacterium tuberculosis*) into *Escherichia coli* strain TUNER (DE3) (Novagen) and cultured to 0.5 OD_600nm_ in LB supplemented with 50 μg/ml kanamycin and 100 μg/ml of Ampicillin at 37 °C and 180 rpm. Expression of the FGE was induced by the addition of 0.2 mg/ml L-arabinose and cells were then cooled to 16 °C for 2 hours. Recombinant sulfatase expression was then induced by the addition of 0.1 mM isopropyl β-D-1-thiogalactopyranoside; cells were cultured for another 16 h at 16 °C and 180 rpm. The next day cells were then centrifuged at 5,000 × g and resuspended in 20 mM HEPES, pH 7.4, with 500 mM NaCl before being sonicated on ice. Recombinant protein was then purified by immobilized metal ion affinity chromatography using a cobalt-based matrix (Talon, Clontech) and, after a wash with resuspension buffer, eluted with a step gradient of 10, 50, and 100 mM imidazole (x2) in resuspension buffer. Proteins were then analysed by SDS-PAGE gel for purity and appropriately pure fractions were concentrated in centrifugal concentrators with a molecular mass cutoff of 30 kDa and further purified by size exclusion in 10 mM HEPES, pH 7.0 with 150 mM NaCl using a 16/60 S200 superdex size exclusion column. Fractions from this were then again subject to SDS-PAGE analysis and fractions judged to >95 % pure pooled and where needed were concentrated in centrifugal concentrators with a molecular mass cutoff of 30 kDa for further downstream structural analyses. Protein concentrations were determined by measuring absorbance at 280 nm using the molar extinction coefficient calculated by ProtParam on the ExPasy server (web.expasy.org/protparam/).

**Coupled assay and Michaelis Menten kinetics**

Coupled assays were performed using 96-well black-wall, clear-bottom, Greiner fluorescence plates, and a BMG Labtech FLUOstar fluorescence spectrophotometer. Primary enzymes (BT1636^3S-Gal^ and BT1622^3S-Gal/GalNAc^) were combined with secondary enzymes (BT0461^GH2^ for Gal substrates and BT4243^GH109^ for GalNAc substrates) and MU-β-3S-Gal (**1**), MU-β-3S-GalNAc (**2**), or MU-α-3S-GalNAc (**3**) as appropriate. Primary enzyme rate determination was confirmed by increasing primary enzyme concentration against a constant secondary enzyme concentration. Michealis-Menten kinetics were measured using 2 μM secondary enzyme, substrate concentration 5 mM - 4 mM, and either 1mM BT1622 (with **2** or **3**), 2.5 mM BT1622^3S-Gal/GalNAc^ (with **1**) or 250 nM BT1636^3S-Gal^ (with **1**). Production of methylumbelliferone was followed at λ_ex/em_ = 355/460 nM, gain 400, and compared to a standard curve prepared from commercial methylumbelliferone (Merck) to determine rates of reaction. Data processing and curve fitting was carried out using GrahpPad Prism.

**IC_50_ assays**

IC_50_ values for metal oxides were calculated from a dilution series of inhibitor compound (2.5 mM to 10 nM) in reactions containing 2 μM secondary enzyme (BT0461^GH2^ or BT4243^GH109^ as above), 250 nM BT1636^3S-Gal^ OR 2.5 μM BT1622^3S-Gal/GalNAc^ (with 1) OR 1 μM BT1622^3S-Gal/GalNAc^ (with 3) and 200 μM MU-β-3S-Gal (1) OR 400 μM MU-β-3S-GalNac (1) as appropriate. The solution of sodium vanadate was boiled just before use to ensure that it was in its monomeric form. Production of methylumbelliferone was followed at λ_ex/em_ = 355/460 nM , gain 400, and compared to a standard curve of methylumbelliferone prepared from commercial material (Merck) to determine rates of reaction. Data processing and curve fitting was carried out using GrahpPad Prism.

**Whole cell/lysate assays**

*Bacteriodes thetaiotamicron* strains *Δtdk* (a genetically manipulatable strain used as wildtype as it displays no auxotrophies), *Δbt1636*, *Δbt1622*, and *Δbt1636Δbt1622Δbt4683* were grown overnight on 5 mL BHI media (Sigma) supplemented with 5 μL histidine/heamatin 0.2 M/1.2 mg ml^-1^. Strains were subcultured into minimal media (Supplemental table S1) supplemented with 5 mg ml^-1^ gastric mucin oligosaccharides. Cultures were grown to mid-exponential phase and half the volume sonicated using a ThermoFisher Dismembrator (1 s on: 5 s off pulse, 30% power). Cells, or lysate, were variously combined with 2 μM secondary enzyme and 200/400 μM MU-β-3S-Gal/MU-β-3S-GalNac as appropriate. Production of methylumbelliferone was followed at λ_ex/em_ = 355/460 nm, gain 400, and compared to a standard curve prepared from commercial methylumbelliferone (Merck) to determine rates of reaction. Data processing and curve fitting was carried out using GraphPad Prism.

**IC_50_ determination using whole cells**

Bacteriodes thetaiotamicron strains *Δ*tdk (a genetically manipulatable strain used as wildtype as it displays no auxotrophies) was grown overnight on 5 mL BHI media (Sigma) supplemented with 5 μL histidine/heamatin 0.2 M/1.2 mg ml-1. Strains were subcultured into minimal media (Supplemental table S1) supplemented with 5 mg ml-1 gastric mucin oligosaccharides. Cultures were grown to mid-exponential phase and combined with 2 μM secondary enzyme and 200 μM MU-β-3S-Gal, as well as a series of concentrations of metal oxide inhibitor (2.5 mM to 10 nM). Production of methylumbelliferone was monitored at λ_ex/em_ = 355/460 nm, gain 400, and compared to a standard curve prepared from commercial methylumbelliferone (Merck) to determine rates of reaction. Data processing and curve fitting was carried out using GraphPad Prism.

**Thin layer chromatography**

*B. theta* *Δtdk* (a genetically manipulatable strain of B. theta which displays no auxotrophies and used as wildtype) was prepared on BHI/histidine/heamatin as above and subcultured into minimal media with 5 mgml^-1^ chondroitin sulphate A (CSA). Cultures were grown to mid-exponential phase and half the volume sonicated using a ThermoFisher Dismembrator (1on:5off s pulse, 30% power). The cells, or lysate, were combined with 5 mM ΔUA-4S-GalNAc in 10 μL volumes, and kept at 37 °C for 1, or 16 hours. The resulting volumes were boiled and spun down before spotting onto aluminium backed silica TLC plates. TLC was carried out using a 2:1:1 solution of butanol:acetic acid:H_2_O and carbohydrates were visualized using a DPA stain (0.3 % HCl, 1.7 % aniline, 8.8 % H_2_PO_3_, 88 % ethyl acetate, 104 mM diphenylamine), with heating at 450 °C using a heatgun.

**High performance anion exchange chromatography**

Degradation of α-Δ4,5-uronic acid-(1🡪3)-4-*O*-sulfo-D-GalNAc (UA-4SGalNAc) by whole cells or sonicated cell lysates was monitored by HPAEC using an ICS-6000 with an attacted VWD, Thermofisher, absorbance detector at *A*_235nm_ to detect the loss of carbon–carbon double bond products. A carbopac PA-200 (3 x 250 mm) with a preceding PA-200 guard column (3 x 50 mm) was used to perform the separation of sugar substrates with H_2_O, pH 3.5, as the eluent A and a second eluent, B, of H_2_O with 3 M NaCl used to generate a linear NaCl gradient to 60% over 40 min, then 100 % NaCl for 10 min before running the column back into H_2_O for 10 min. All enzymatic assays were performed in biological triplicate.

**Crystallographic methods**

Protein crystals of BT1636S (recombinantly expressed as above, 30 mg/ml) were prepared in a vapour diffusion experiment using 40 % MPD, 5 % PEG 8000, and 100 mM sodium cacodylate pH 6.5 as reservoir solution. Broad, plate-form crystals were soaked for 24 hours using reservoir solution adulterated with 10 mM sodium salt of phosphate, chromate, and molybdate before harvesting. Diffraction was carried out using Diamond Light Source UDC ‘ligand’ strategy, and scaled data from xia2 3dii was further processed with AIMLESS^[1]^, DIMPLE^[2]^, Phaser^[3]^, Refmac5^[4]^, Servalcat^[5]^ and COOT^[6]^. Crystallographic figures were prepared using Pymol.

# Chemical Synthesis

## General experimental procedures

Reagents and solvents were purchased from commercial sources and used without further purification unless stated otherwise. Anhydrous solvents were dried and stored over activated 3 Å molecular sieves under a N_2_ atmosphere. All reactions were performed under a N_2_ atmosphere unless stated otherwise. The progression of the reaction was monitored by TLC analysis using Merck Millipore Supelco aluminum sheets (silica gel 60 F254) and visualized by UV detection (254 nm), and by spraying with cerium molybdate spray (solution of 25 g/L (NH_4_)_6_Mo_7_O_24_⋅4H_2_O and 10 g/L (NH_4_)_4_Ce(SO_4_)_4_⋅2H_2_O in 10% aq. sulfuric acid) followed by charring at ± 200°C. TLC-MS analysis was performed on a Camag TLC-MS Interface coupled to an API165 (SCIEX) mass spectrometer (eluted with tert-butyl methyl ether/EtOAc/MeOH, 5:4:1, v/v/v + 0.1% formic acid, flow rate 0.12 mL/min). Flash column chromatography was carried out using Macherey-Nagel silica gel 60 Å (40-63 μm). Automated flash column chromatography was performed on Biotage®Isolera™Systems or Biotage®Selekt System using a linear gradient of eluents on pre-packed silica cartridges purchased from Screening Devices B.V. (Ultrapure Irregular Silica Gel, 40–63 μm, 60 Å). The sodium-form Dowex™ 50WX4 was prepared from the DOWEX 50WX8 hydrogen form by thoroughly rinsing with 1 M NaOH, followed by 1 M NaHCO_3_ (stored over 0.1 M aq. NaHCO_3_). The ammonium-form Dowex™ 50WX4 was prepared from the DOWEX 50WX8 hydrogen form by dissolving it in a 30% aq. NH_3_ solution, followed by thoroughly rinsing with 0.1 M NH_4_OAc. Both resins were rinsed thoroughly with water prior to use. ^1^H and ^13^C spectra were recorded on a Bruker AV-400 (400 MHz), Bruker AV-400 Wide Bore (400 MHz), and a Bruker AV-500 (500 MHz) instrument. Deuterated chloroform was stored over K_2_CO_3_ and activated 3 Å molecular rods (size 1/16 in., Sigma Aldrich). Coupling constants (*J*) are given in hertz (Hz) and chemical shifts (δ) are reported in parts per million (PPM) relative to the residual signal of the solvent, or to tetramethylsilane for CDCl_3_. All given ^13^C APT spectra are proton decoupled, and are presented with even signals (C_q_ and CH_2_) as positive and odd signals (CH and CH_3_) as negative. HH-COSY and HSQC spextra were used to accomplish the structural assignments. The stereochemical outcome of the product was determined by examining the coupling constants (^3^J_H-H_) of the corresponding signal. ^1^H and ^13^C signal assignment was done in accordance with IUPAC nomenclature. High-resolution mass spectra (HRMS) were recorded by direct injection on an LTQ Orbitrap (Thermo Finnigan) mass spectrometer equipped with an electrospray ion source (positive ion mode, source voltage 3.5 kV, sheath gas flow 10, capillary temperature 250 °C). Spectra were collected at a resolution of R = 60 000 at m/z = 400 (mass range m/z 150–4000) using dioctyl phthalate (m/z = 391.28428) as an internal lock mass.

## Synthesis of MU-β-3S-Gal

**4-Methylumbelliferyl 2,3,4,6-tetra-*O*-acetyl-β-D-galactopyranoside (5)**

Commercially available β-D-galactose pentaacetate (19.5 g, 50.0 mmol, 1.0 equiv.) was dissolved in anhydrous DCM (25 mL, 2 M) and cooled on ice. A solution of HBr in acetic acid (33 wt. %; 18.0 mL, 100 mmol, 2.0 equiv.) was added, and the mixture was stirred for 2 h at 0 °C. After full conversion was observed (TLC: R*_f_* = 0.5, EtOAc/pentane 4:6 v/v), the reaction mixture was poured into a separatory funnel containing ice-water and DCM (200 mL). The organic layer was washed with ice-water until neutral pH, then with brine, dried over Na₂SO₄, and filtered. The resulting glycosyl bromide in DCM (300 mL, 0.17 M) was added to a suspension of TBAHS (17.0 g, 50.0 mmol, 1.0 equiv.) and 4-methylumbelliferone (26.4 g, 150 mmol, 3.0 equiv.) in water (200 mL, 0.25 M). NaOH (8.00 g, 200 mmol, 4.0 equiv.) was added and the mixture was stirred overnight at room temperature. Upon full conversion (TLC: R*_f_* = 0.2, EtOAc/pentane 4:6 v/v), the mixture was further diluted with 1 M NaOH and the aqueous layer was extracted with DCM (3 x). The combined organic layers were washed with brine, dried over MgSO_4_, filtered and concentrated under reduced pressure. The crude product was purified by flash column chromatography (SiO_2_, dry loading on Celite®; 20→80% EtOAc/pentane), yielding the title compound (20.9 g, 39.5 mmol, 79% over two steps) as a clear brittle foam. Analytical data are in agreement with the literature precedent^[7]^. **^1^H NMR** (400 MHz, CDCl_3_, HH-COSY, HSQC): δ 7.53 (d, *J* = 8.7 Hz, 1H, CH_arom_), 6.98 (d, *J* = 2.4 Hz, 1H, CH_arom_), 6.95 (dd, *J* = 8.7, 2.5 Hz, 1H, CH_arom_), 6.20 (q, *J* = 1.2 Hz, 1H, H-3’ MU), 5.53 (dd, *J* = 10.4, 7.9 Hz, 1H, H-2), 5.49 (dd, *J* = 3.5, 1.1 Hz, 1H, H-4), 5.18 – 5.11 (m, 2H, H-3, H-1), 4.26 – 4.18 (m, 2H, H-6), 4.12 (ddd, *J* = 7.5, 5.5, 1.2 Hz, 1H, H-5), 2.42 (d, *J* = 1.3 Hz, 3H, CH_3_ MU), 2.20 (s, 3H, CH_3_ Ac), 2.12 (s, 3H, CH_3_ Ac), 2.08 (s, 3H, CH_3_ Ac), 2.03 (s, 3H, CH_3_ Ac); **^13^C NMR** (101 MHz, CDCl_3_, HSQC): δ 170.6 (C_q_), 170.3 , 170.2, 169.5, 160.9, 159.4, 155.0, 152.3, 125.9 (CH_arom_), 115.7 (C_q_), 114.1 (CH_arom_), 113.4 (C-3’ MU), 104.2 (CH_arom_), 99.1 (C-1), 71.7 (C-5), 70.8 (C-3), 68.5 (C-2), 66.9 (C-4), 61.6 (C-6), 20.9 (CH_3_ Ac), 20.8, 20.8, 20.7, 18.8 (CH_3_ MU); **ESI** **HRMS**: [M+Na^+^] calculated for: C_24_H_26_NaO_12_ 529.1316; found 529.1317.

**4-Methylumbelliferyl-β-D-galactopyranoside (6)**

To a solution of peracetylated compound **5** (20.9 g, 39.5 mmol, 1.0 equiv.) in MeOH (400 mL, 0.1 M) was added sodium methoxide (427 mg, 7.91 mmol, 0.2 equiv.). The solution was stirred for 3 h, after which full conversion was observed (TLC: R*_f_* = 0.6, MeOH/DCM 2:8 v/v). Subsequently, the suspension was quenched with AcOH until pH 5 was reached, and the white solid was collected by filtration to afford the title compound (13.2 g, 39.1 mmol, 99%) as a white powder. Analytical data are in agreement with the literature precedent.^[7]^ **^1^H NMR** (400 MHz, DMSO, HH-COSY, HSQC): δ 7.70 (d, *J* = 8.9 Hz, 1H, CH_arom_), 7.08 – 6.98 (m, 2H, CH_arom_), 6.24 (q, *J* = 1.3 Hz, 1H, H-3’ MU), 5.28 (d, *J* = 4.8 Hz, 1H, 3-OH), 5.06 – 4.88 (m, 2H, H-1, 2-OH), 4.71 (t, *J* = 5.5 Hz, 1H, 6-OH), 4.59 (d, *J* = 4.5 Hz, 1H, 4-OH), 3.76 – 3.64 (m, 2H, H-4, H-5), 3.64 – 3.41 (m, 4H, H-2, H-3, H-6), 2.40 (d, *J* = 1.3 Hz, 3H, CH_3_ MU); **^13^C NMR** (101 MHz, DMSO, HSQC): δ 160.2 (C_q_), 160.1, 154.4, 153.4, 126.4 (CH_arom_), 114.0 (C_q_), 113.4 (CH_arom_), 111.6 (C-3’ MU), 103.1 (CH_arom_), 100.6 (C-1), 75.7 (C-5), 73.2 (C-3), 70.1 (C-2), 68.1 (C-4), 60.4 (C-6), 18.1(CH_3_ MU); **ESI** **HRMS**: [M+Na^+^] calculated for: C_16_H_18_NaO_8_ 361.0894; found 361.0892.

**Ammonium 4-methylumbelliferyl-3-*O*-sulfo-β-D-galactopyranoside (1)**

A round-bottom flask was equipped with a magnetic stirring bar and a Dean–Stark apparatus. 4-Methylumbelliferyl-β-D-galactopyranoside **6** (62.2 mg, 184 μmol, 1.0 equiv.) was added and dissolved in a mixture of anhydrous DMF and toluene (1:2 v/v, 4 mL, 0.05 M). Subsequently, dibutyltinoxide (57 mg, 229 μmol, 1.2 equiv.) was added, and the reaction mixture was refluxed for 2 h. The solvent was then removed under reduced pressure. The crude intermediate was dissolved in anhydrous DMF (2 mL, 0.1 M) and SO_3_·Et_3_N (47 mg, 260 μmol, 1.4 equiv.) was added. The reaction mixture was stirred overnight at room temperature, after which full conversion was observed (TLC: R*_f_* = 0.4, MeOH/DCM 2:8 v/v). The solvent was removed *in vacuo*, and the residue was purified by automated flash column chromatography (SiO_2_, dry loading on Celite®; 5→25% MeOH/DCM), followed by NH_4_^+^-Dowex™ 50WX4 ion exchange and lyophilization to afford compound **1** as a white powder (44.8 mg, 103 μmol, 57% over 2 steps). **^1^H NMR** (400 MHz, MeOD, HH-COSY, HSQC): δ 7.71 (d, *J* = 8.8 Hz, 1H, CH_arom_), 7.13 (dd, *J* = 8.7, 2.4 Hz, 1H, CH_arom_), 7.10 (d, *J* = 2.4 Hz, 1H, CH_arom_), 6.20 (q, *J* = 1.3 Hz, 1H, H-3’ MU), 5.13 (d, *J* = 7.7 Hz, 1H, H-1), 4.38 (dd, *J* = 9.6, 3.2 Hz, 1H, H-3), 4.34 (dd, *J* = 3.2, 0.9 Hz, 1H, H-4), 4.01 (dd, *J* = 9.6, 7.7 Hz, 1H, H-2), 3.87 – 3.71 (m, 3H, H-5, H-6), 2.46 (d, *J* = 1.2 Hz, 3H, CH_3_ 4 MU); **^13^C NMR** (101 MHz, MeOD, HSQC): δ 163.4 (C_q_), 162.0, 156.0, 155.5, 127.3 (CH_arom_), 116.0 (C_q_), 115.1 (CH_arom_), 112.8 (C-3’ MU), 105.0 (CH_arom_), 102.1 (C-1), 81.7 (C-5), 76.9 (C-3), 70.4 (C-2), 68.3 (C-4), 62.3 (C-6), 18.7 (CH_3_ MU); **ESI** **HRMS**: [M+H^+^] calculated for: C_16_H_19_O_11_S 419.0647; found 419.0643.

## Synthesis of MU-β-3S-GalNAc

**2-Acetamido-1,3,4,6-tetra-*O*-acetyl-2-deoxy-β-D-galactopyranoside (7)**

To an ice-cold solution of commercially available D-galactosamine hydrochloride (3.24 g, 15 mmol, 1.0 equiv.) in anhydrous pyridine (30 mL, 0.5 M) was added dropwise acetic anhydride (21 mL, 225 mmol, 15 equiv.). The reaction mixture was allowed to warm-up to room temperature and was stirred for 3 days. Upon full conversion (TLC: R*_f_* = 0.3, MeOH/DCM 2:8 v/v), the reaction mixture was then poured into ice-water, and the resulting suspension was stirred for 1 h. The precipitate was collected by vacuum filtration and dried *in vacuo* to afford the title compound (4.86 g, 12.5 mmol, 83%) as a white powder. **^1^H NMR** (400 MHz, DMSO, HH-COSY, HSQC): δ 7.91 (d, J = 9.2 Hz, 1H, NH), 5.64 (d, J = 8.8 Hz, 1H, H-1), 5.26 (dd, J = 3.4, 1.2 Hz, 1H, H-4), 5.06 (dd, J = 11.3, 3.4 Hz, 1H, H-3), 4.22 (app. ddd, J = 6.8, 5.6, 1.2 Hz, 1H, H-5), 4.14 – 3.95 (m, 3H, H-2, H-6), 2.12 (s, 3H, CH_3_ Ac), 2.03 (s, 3H, CH_3_ Ac), 1.99 (s, 3H, CH_3_ Ac) 1.90 (s, 3H, CH_3_ Ac), 1.78 (s, 3H CH_3_ Ac); **^13^C NMR** (101 MHz, DMSO, HSQC): δ 170.0 (C_q_), 169.9, 169.6, 169.6, 169.0, 92.5 (C-1), 70.8 (C-5), 70.0 (C-3), 66.4 (C-4), 61.4 (C-6), 48.2 (C-2), 22.7 (CH_3_ Ac), 20.6, 20.5, 20.4; **ESI** **HRMS**: [M+Na^+^] calculated for: C_16_H_23_NNaO_10_ 412.1214; found 412.1219.

**4-Methylumbelliferyl 2-acetamido-3,4,6-tri-*O*-acetyl-2-deoxy-β-D-galactopyranoside (8)**

Peracetylated compound **7** (367 mg, 0.94 mmol, 1.0 equiv.) was dissolved in acyl chloride (10 mL, 0.1 M). A solution of HCl in dioxane (4 M; 112 μL, 0.47 mmol, 0.5 equiv.) was added and the reaction mixture was stirred overnight at 40 °C. Upon full conversion (TLC: R*_f_* = 0.4, EtOAc/pentane 7:3 v/v), the reaction was carefully quenched with sat. aq. NaHCO_3_. The suspension was poured into a separatory funnel containing DCM and the layers were separated. The organic layer was subsequently washed with H_2_O (3 x), sat. aq. NaHCO_3_ (3 x), and brine, dried over Na_2_SO_4_, filtered, and concentrated *in vacuo*. The resulting anomeric chloride was dissolved in DCM (6 mL, 0.16 M) and added dropwise to a mixture of 4-methylumbelliferone (248 mg, 1.41 mmol, 1.5 equiv.), TBAB (454 mg, 1.41 mmol, 1.5 equiv.) and CsOH (50 wt. % in water; 229 μL, 1.32 mmol, 1.4 equiv.) in water (4 mL, 0.24 M). The suspension was stirred for 1 h, after which full conversion was observed (TLC: R*_f_* = 0.1, EtOAc/pentane 7:3 v/v). The organic and aqueous layers were separated and the organic layer was washed with water (2 x), dried over MgSO_4_, filtered, and concentrated under reduced pressure. The crude product was purified by automated flash column chromatography (SiO_2_, dry loading on Celite®; 50→100% EtOAc/pentane) to afford MU compound **8** (166 mg, 0.34 mmol, 35% over 2 steps) as a white powder. **^1^H NMR** (400 MHz, MeOD, HH-COSY, HSQC): δ 7.71 (d, *J* = 8.6 Hz, 1H, CH_arom_), 7.07 – 6.99 (m, 2H, CH_arom_), 6.22 (q, *J* = 1.2 Hz, 1H, H-3’ 4MU), 5.45 (dd, *J* = 3.3, 1.1 Hz, 1H, H-4), 5.35 (d, *J* = 8.5 Hz, 1H, H-1), 5.23 (dd, *J* = 11.2, 3.4 Hz, 1H, H-3), 4.40 (dd, *J* = 11.2, 8.5 Hz, 1H, H-2), 4.32 (app. ddd, *J* = 6.8, 5.5, 1.2 Hz, 1H, H-5), 4.19 (dd, *J* = 6.4, 2.5 Hz, 2H, H-6), 2.45 (d, *J* = 1.2 Hz, 3H, CH_3_ MU), 2.18 (s, 3H, CH_3_ Ac), 2.07 (s, 3H, CH_3_ Ac), 1.99 (s, 3H, CH_3_ Ac), 1.95 (s, 3H, CH_3_ Ac); **^13^C NMR** (101 MHz, MeOD, HSQC) δ 173.9 (C_q_), 172.1, 172.0, 171.6, 163.0, 161.3, 156.0, 155.3, 127.4 (CH_arom_), 116.4 (C_q_), 115.1 (CH_arom_), 113.2 (C-3’ MU), 104.7 (CH_arom_), 99.8 (C-1), 72.5 (C-5), 71.8 (C-3), 68.1 (C-4), 62.9 (C-6), 51.3 (C-2), 22.8 (CH_3_ Ac), 20.6, 20.5, 18.6 (CH_3_ MU); **ESI** **HRMS**: [M+Na^+^] calculated for: C_24_H_27_NNaO_11_ 528.1476; found 528.1481.

**4-Methylumbelliferyl-2-acetamido-2-deoxy-β-D-galactopyranoside (9)**

Peracetylated compound **8** (162 mg, 0.32 mmol, 1.0 equiv.) was dissolved in anhydrous MeOH (2 mL, 0.16 M) and NaOMe (4 M in MeOH; 16 μL, 64 μmol, 0.2 equiv.) was added. The solution was stirred for 3 h, after which full conversion was observed (TLC: R*_f_* = 0.3, MeOH/DCM 2:8 v/v). Subsequently, the suspension was quenched with AcOH until pH 5 was reached, and the solid was collected by filtration to afford triol **9** (102 mg, 0.27 mmol, 85%) as a white powder. Analytical data are in agreement with the literature precedent^[8]^. **^1^H NMR** (400 MHz, DMSO, HH-COSY, HSQC): δ 7.78 (d, *J* = 9.1 Hz, 1H, NH), 7.69 (d, *J* = 8.7 Hz, 1H, CH_arom_), 7.00 (d, *J* = 2.4 Hz, 1H, CH_arom_), 6.95 (dd, *J* = 8.7, 2.4 Hz, 1H CH_arom_), 6.25 (d, *J* = 1.4 Hz, 1H, H-3’ MU), 5.09 (d, *J* = 8.4 Hz, 1H, H-1), 4.81 (d, *J* = 6.3 Hz, 1H, 3-OH), 4.74 – 4.69 (m, 2H, 4-OH, 6-OH), 4.02 (app. dt, *J* = 10.5, 8.7 Hz, 1H, H-2), 3.73 (app. t, *J* = 3.8 Hz, 1H, H-4), 3.66 – 3.49 (m, 4H, H-6, H-3, H-5), 2.40 (d, *J* = 1.2 Hz, 3H, CH_3_ MU), 1.80 (s, 3H, CH_3_ Ac); **^13^C NMR** (101 MHz, DMSO, HSQC): δ 169.6 (C_q_), 160.2, 160.1, 154.4, 153.3, 126.5 (CH_arom_), 114.2 (C-3’ MU), 113.6 (CH_arom_), 111.8, 103.2, 99.1 (C-1), 75.9 (C-5), 71.1 (C-3), 67.5 (C-4), 60.5 (C-6), 51.8 (C-2), 23.1 (CH_3_ Ac), 18.2 (CH_3_ MU); **ESI** **HRMS**: [M+Na^+^] calculated for: C_18_H_21_NNaO_8_ 402.1159; found 402.1162.

**Sodium 4-methylumbelliferyl-2-acetamido-2-deoxy-3-*O*-sulfo-β-D-galactopyranoside (2)**

A round-bottom flask was equipped with a magnetic stir bar and a Dean–Stark apparatus. Triol **9** (96.2 mg, 255 μmol, 1.0 equiv.) was added and dissolved in a mixture of anhydrous DMF and toluene (1:2 v/v, 5 mL, 0.05 M). Bu_2_SnO (72.2 mg, 290 μmol, 1.2 equiv.) was added, and the reaction mixture was refluxed for 3 h. The solvent was then removed under reduced pressure. The crude intermediate was dissolved in anhydrous DMF (2.5 mL, 0.1 M) and SO_3_·Et_3_N (60.0 mg, 331 μmol, 1.3 equiv.) was added. The reaction mixture was stirred overnight at room temperature, after which full conversion was observed (TLC: R*_f_* = 0.2, MeOH/DCM 2:8 v/v). The solvent was removed *in vacuo*, and the residue was purified by automated flash column chromatography (SiO_2_, dry loading on Celite®; 10→35% MeOH/DCM), followed by Na^+^-Dowex® 50WX4 ion exchange and lyophilization to afford compound **2** as a white powder (18.5 mg, 38.5 μmol, 15% over 2 steps). **^1^H NMR** (500 MHz, MeOD, HH-COSY, HSQC): δ 7.70 (d, *J* = 8.6 Hz, 1H, CH_arom_), 7.09 – 7.01 (m, 2H, CH_arom_), 6.20 (t, *J* = 1.3 Hz, 1H, H-3’ MU), 5.31 (d, *J* = 8.3 Hz, 1H, H-1), 4.51 (dd, *J* = 11.0, 3.1 Hz, 1H, H-3), 4.39 (dd, *J* = 11.1, 8.3 Hz, 1H, H-2), 4.36 (d, *J* = 3.1 Hz, 1H, H-4), 3.83 – 3.77 (m, 3H, H-5, H-6), 2.45 (d, *J* = 1.2 Hz, 3H, CH_3_ MU), 1.97 (s, 3H, CH_3_ Ac); **^13^C NMR** (126 MHz, MeOD, HSQC): δ 174.1 (C_q_), 163.3, 161.9, 156.0, 155.5, 127.3 (CH_arom_), 116.1 (C_q_), 115.1 (CH_arom_), 112.9 (C-3’ MU), 104.9 (CH_arom_), 100.7 (C-1), 78.5 (C-3), 77.0 (C-5), 67.8 (C-4), 62.4 (C-6), 51.9 (C-2), 23.1 (CH_3_ Ac), 18.6 (CH_3_ MU); **ESI** **HRMS**: [M+H^+^] calculated for: C_18_H_22_NO_11_ 458.0763; found 458.0765.

## Synthesis of MU-α-3S-GalNAc

Scheme S1. Synthesis of sulfated MU-glycosides. *Reagents and conditions*: a) diphenyl diselenide, (diacetoxyiodo)benzene, trimethylsilyl azide, DCM, −30 °C → −20 °C (48%); b) *i*. NaOMe, MeOH, rt; *ii*. di-tert-butylsilyl bis(trifluoromethanesulfonate), pyridine, −30 °C → −20 °C (89% over two steps).

**Phenyl 3,4,6-tri-*O*-acetyl-2-azido-2-deoxy-1-seleno-α-D-galactopyranoside (S1)**

Commercially available tri-*O*-acetyl-D-galactal (5.99 g, 21.9 mmol, 1.0 equiv.) was dissolved in anhydrous DCM (100 mL, 0.2 M), and diphenyl diselenide (6.85 g, 21.9 mmol, 1.0 equiv.) was added. The reaction mixture was cooled to −30 °C, and (Diacetoxyiodo)benzene (7.07 g, 21.9 mmol, 1.0 equiv.) and trimethylsilyl azide (5.80 mL, 43.9 mmol, 2.0 equiv.) were added. The reaction mixture was stirred overnight at −20 °C. After full conversion was observed (TLC: R*_f_* = 0.7, EtOAc/pentane 3:7 v/v), the reaction was quenched with cyclohexene, and the solvent was removed under reduced pressure. The crude product was purified by automated flash column chromatography (SiO_2_; 5 → 30% EtOAc/pentane) to remove lipophilic byproducts, followed by recrystallization from hot EtOH to afford azide **S1** (4.99 g, 10.6 mmol, 48%) as a white powder. Analytical data are in agreement with the literature precedent^[9]^. **^1^H NMR** (400 MHz, CDCl_3_, HH-COSY, HSQC): δ 7.63 – 7.57 (m, 2H, CH_arom_), 7.34 – 7.26 (m, 3H, CH_arom_), 6.00 (d, *J* = 5.4 Hz, 1H, H-1), 5.47 (dd, *J* = 3.3, 1.3 Hz, 1H, H-4), 5.11 (dd, *J* = 10.9, 3.2 Hz, 1H, H-3), 4.66 (ddd, *J* = 7.2, 5.6, 1.4 Hz, 1H, H-5), 4.26 (dd, *J* = 10.8, 5.4 Hz, 1H, H-2), 4.09 – 3.99 (m, 2H, H-6), 2.15 (s, 3H, CH_3_ Ac), 2.06 (s, 3H, CH_3_ Ac), 1.97 (s, 3H, CH_3_ Ac); **^13^C NMR** (101 MHz, CDCl_3_, HSQC): δ 170.4 (C_q_), 170.0, 169.7, 134.9 (t, *J* = 5.3 Hz, CH_arom_), 129.3 (CH_arom_), 128.3, 127.6 (C_q_), 84.2 (t, *J* = 43.0 Hz, H-1), 71.3 (C-3), 69.0 (C-5), 67.2 (C-4), 61.6 (C-6), 58.8 (C-2), 20.8 (CH_3_ Ac), 20.7; **ESI** **HRMS**: [M+Na^+^] calculated for: C_18_H_21_N_3_NaO_7_Se 494.0438; found 494.0434.

**Phenyl 2-azido-2-deoxy-4,6-*O*-(di-*tert*-butylsilylene)-1-seleno-α-D-galactopyranoside (11)**

Peracetylated compound **S1** (4.99 g, 10.6 mmol, 1.0 equiv.) was dissolved in MeOH (100 mL, 0.1 M) and NaOMe (115 mg, 2.12 mmol, 0.2 equiv.) was added. The solution was stirred overnight, after which full conversion was observed (TLC: R*_f_* = 0.1, EtOAc/pentane 3:7 v/v). The reaction was quenched with Amberlite™ IR-120 resin (H^+^ form), until pH 5 was reached. The solvent was removed under reduced pressure, followed by co-evaporation with anhydrous CHCl_3_ to remove residual MeOH. The resulting crude mixture was dissolved in anhydrous DMF (50 mL, 0.2 M) and cooled to −30 °C. Subsequently, di-tert-butylsilyl bis(trifluoromethanesulfonate) (3.6 mL, 11.1 mmol, 1.05 equiv) was added, and the mixture was stirred for 10 min at −20 to −30 °C, after which anhydrous pyridine (1.7 mL, 21.2 mmol, 2.0 equiv) was added. The reaction mixture was stirred for 1 h at the same temperature. After full conversion was observed (TLC: R*_f_* = 0.7, Et_2_O/pentane 2:8 v/v), the reaction was diluted with Et_2_O and quenched with sat. aq. NaHCO_3_ . The organic layer was washed with H_2_O (2 x), dried over MgSO_4_, filtered, and evaporated under reduced pressure. Automated flash column chromatography (SiO_2_, dry loading on Celite®; 1 → 20% Et_2_O/pentane) afforded silylidene **11** (4.55 g, 9.40 mmol, 89% over 2 steps) as a clear foam. Analytical data are in agreement with the literature precedent^[10]^. **^1^H NMR** (400 MHz, CDCl_3_, HH-COSY, HSQC): δ 7.59 – 7.51 (m, 2H, CH_arom_), 7.33 – 7.26 (m, 3H, CH_arom_), 5.93 (d, *J* = 5.2 Hz, 1H, H-1), 4.49 (dd, *J* = 3.4, 1.2 Hz, 1H, H-4), 4.30 (dd, *J* = 12.7, 2.3 Hz, 1H, H-6), 4.20 (app. ddt, *J* = 2.4, 1.6, 0.7 Hz, 1H, H-5), 4.07 – 3.99 (m, 2H, H-2, H-6), 3.79 (app. td, *J* = 10.4, 3.4 Hz, 1H, H-3, H-3), 2.76 (d, *J* = 10.8 Hz, 1H, 3-OH), 1.08 – 0.98 (m, 18H, SiC(CH_3_)_3_); **^13^C NMR** (101 MHz, CDCl_3_, HSQC): δ 134.5 (t, *J* = 4.8 Hz, CH_arom_), 129.3 (CH_arom_), 128.5 (C_q_), 128.0 (CH_arom_), 85.5 (t, *J* = 41.8 Hz, H-1), 72.4 (C-4), 71.9 (C-3), 69.9 (C-5), 66.8 (C-6), 62.2 (C-2), 27.7 (SiC(*C*H_3_)_3_), 27.4, 23.5 (Si*C*(CH_3_)_3_), 20.9; **ESI** **HRMS**: [M+Na^+^] calculated for: C_20_H_31_N_3_NaO_4_SeSi 508.1142; found 508.1143.

**Phenyl 2-azido-2-deoxy-3-*O*-acetyl-4,6-*O*-(di-*tert*-butylsilylene)-1-seleno-α-D-galactopyranoside (12)**

Alcohol **11** (4.55 g, 9.40 mmol, 1.0 equiv.) was dissolved in anhydrous pyridine (40 mL, 0.4 M) and cooled on ice. Ac_2_O (1.8 mL, 18.8 mmol, 2.0 equiv.) was added dropwise, and the reaction mixture was allowed to warm to room temperature and stirred overnight. Upon full conversion (TLC: R*_f_* = 0.8, Et_2_O/pentane 1:9 v/v), the reaction was quenched with MeOH on ice. The mixture was poured into a separatory funnel containing Et_2_O and sat. aq. NaHCO_3_. The aqueous layer was extracted with Et_2_O (3 x) and the combined organic layers were washed with brine, dried over MgSO_4_, filtered, and concentrated *in vacuo*. Purification of the crude product with automated flash column chromatography (SiO_2_, dry loading on Celite®; 1 → 10% Et_2_O/pentane) afforded fully protected glycosyl donor **12** (4.74 g, 9.00 mmol, 96%) as a white powder. **^1^H NMR** (400 MHz, CDCl_3_, HH-COSY, HSQC): δ 7.59 – 7.52 (m, 2H, CH_arom_), 7.33 – 7.26 (m, 3H, CH_arom_), 5.97 (d, *J* = 5.3 Hz, 1H, H-1), 4.87 (dd, *J* = 10.6, 3.0 Hz, 1H, H-3), 4.76 (dd, *J* = 3.1, 1.1 Hz, 1H, H-4), 4.41 (dd, *J* = 10.6, 5.3 Hz, 1H, H-2), 4.25 (dd, *J* = 12.7, 2.3 Hz, 1H, H-6), 4.16 (dq, *J* = 2.0, 1.2 Hz, 1H, H-5), 3.99 (dd, *J* = 12.7, 1.6 Hz, 1H, H-6), 2.17 (s, 3H, CH_3_ Ac), 1.02 (d, *J* = 7.0 Hz, 18H, SiC(CH_3_)_3_); **^13^C NMR** (101 MHz, CDCl_3_, HSQC): δ 170.4 (C_q_), 134.7 (CH_arom_), 129.3, 128.3 (C_q_), 128.1 (CH_arom_), 85.3 (C-1), 74.4 (C-3), 69.7 (C-4, 5), 66.9 (C-6), 58.4 (C-2), 27.7 (SiC(*C*H_3_)_3_), 27.4, 23.4 (Si*C*(CH_3_)_3_), 21.0 (CH_3_ Ac), 20.8 (Si*C*(CH_3_)_3_); **ESI** **HRMS**: [M+Na^+^] calculated for: C_22_H_33_N_3_NaO_5_SeSi 550.1248; found 550.1249.

**2-azido-2-deoxy-3-*O*-acetyl-4,6-*O*-(di-*tert*-butylsilylene)-α/β-D-galactopyranoside (13)**

Selenoglycoside **12** (350 mg, 0.66 mmol, 1.0 equiv.) was dissolved in a mixture of acetone/H_2_O (1:1 v/v; 7 mL, 0.1 M). *N*-Iodosuccinimide (195 mg, 0.87 mmol, 1.3 equiv.) was added and the mixture was stirred for 1 h. Upon full conversion (TLC: R*_f_* = 0.7, R*_f_* = 0.5, Et_2_O/pentane 1:1 v/v), the reaction was quenched with sat. aq. Na_2_S_2_O_3_ and diluted with EtOAc. The organic layer was washed with water, sat. aq. NaHCO_3_, dried over MgSO_4_, filtered, and concentrated under reduced pressure. Automated flash column chromatography (SiO_2_; 10 → 50% Et_2_O/pentane) afforded hemiacetal **13** (225 mg, 0.58 mmol, 88%) as a white powder. Data of the major stereoisomer (α-anomer): **^1^H NMR** (400 MHz, CDCl_3_, HH-COSY, HSQC): δ 5.39 (d, *J* = 3.5 Hz, 1H, H-1α), 5.14 (dd, *J* = 10.8, 2.9 Hz, 1H, H-3α), 4.71 (dd, *J* = 3.0, 1.1 Hz, 1H, H-4α), 4.30 – 4.18 (m, 2H, H-6α), 4.13 (dd, *J* = 12.7, 1.7 Hz, 1H, H-6α), 4.01 (app. q, *J* = 1.7 Hz, 1H, H-5α), 3.92 (dd, *J* = 10.8, 3.5 Hz, 1H, H-2α), 3.14 (bs, 1H, OH-1α), 2.17 (d, *J* = 2.7 Hz, 3H, CH_3_ Ac), 1.08 – 0.98 (m, 18H, SiC(CH_3_)_3_)); **^13^C NMR** (101 MHz, CDCl_3_, HSQC): δ 170.7 (C_q_), 92.6 (C-1α), 71.6 (C-3α), 70.3 (C-4α), 67.1 (C-5α), 67.1 (C-6α), 57.7 (C-2α), 27.6 (SiC(*C*H_3_)_3_), 27.6, 23.3 (Si*C*(CH_3_)_3_), 21.0 (CH_3_ Ac); Diagnostic signals of the minor stereoisomer (β-anomer): **^1^H NMR** (400 MHz, CDCl_3_, HH-COSY, HSQC): δ 4.64 (d, *J* = 8.0 Hz, 1H, H-1β), 4.59 (dd, *J* = 3.2, 1.0 Hz, 1H, H-4β), 4.55 (dd, *J* = 10.5, 3.1 Hz, 1H, H-3β), 3.79 (dd, *J* = 10.5, 8.0 Hz, 1H, H-2β), 3.58 (bs, 1H, OH-1β), 3.46 (app. dt, *J* = 2.1, 1.3 Hz, 1H, H-5β); **^13^C NMR** (101 MHz, CDCl_3_, HSQC): 96.5 (C-1β), 74.4 (C-3β), 71.5 (C-5β), 69.2 (C-4β), 66.9 (C-6β), 61.7 (C-2β), 27.6 (SiC(*C*H_3_)_3_), 27.4, 20.9 (CH_3_ Ac), 20.8 (Si*C*(CH_3_)_3_); **ESI** **HRMS**: [M+Na^+^] calculated for: C_16_H_29_N_3_NaO_6_Si 410.1718; found 410.1724.

**4-Methylumbelliferyl-2-azido-2-deoxy-3-*O*-acetyl-4,6-*O*-(di-*tert*-butylsilylene)-α-D-galactopyranoside (15)**

Hemiacetal **13** (196 mg, 0.51 mmol, 1.0 equiv.) was dissolved in a mixture of acetone/water (50:1 v/v; 5.0 mL, 0.1 M) and cooled on ice. Cs_2_CO_3_ (264 mg, 0.81 mmol, 1.6 equiv.) and 2,2,2-trifluoro-*N*-phenylacetimidoyl chloride (269 mg, 0.81 mmol, 1.6 equiv.) were added, and the reaction mixture was allowed to warm to room temperature and stirred overnight. After full conversion was observed (TLC: major: R*_f_* = 0.8, minor: R*_f_* = 0.3, Et_2_O/pentane 1:9 v/v), the reaction mixture was poured into a separatory funnel containing Et_2_O and sat. aq. NH_4_Cl. The aqueous layer was extracted with Et_2_O (3 x) and the combined organic layers were washed with brine, dried over MgSO_4_, filtered, and concentrated *in vacuo*. The crude product was purified by automated flash column chromatography (SiO_2_, dry loading on Celite®; 1 → 30% Et_2_O/pentane) to obtain imidate  **14** (274 mg, 0.49 mmol, 97%) as an anomeric mixture, which was immediately used in the next reaction.

To imidate **14** (169 mg, 0.30 mmol, 1.0 equiv.) was added 4-methylumbelliferone (106 mg, 0.60 mmol, 2.0 equiv.), and the mixture was co-evaporated with anhydrous toluene (3 x) under an argon atmosphere. The residue was dissolved in anhydrous DCM (3 mL, 0.1 M) and cooled to −25 °C. Trimethylsilyl trifluoromethanesulfonate (11 μL, 59 μmol, 0.2 equiv.) was added and the reaction mixture was allowed to warm to room temperature and stirred for 4 h. Upon full conversion (TLC: R*_f_* = 0.5 Et_2_O/pentane 1:1 v/v) the reaction was quenched with Et_3_N and diluted with sat. aq. NaHCO_3_. The aqueous layer was extracted with DCM (2 x) and the combined organic layers were washed with brine, dried over MgSO_4_, filtered, and concentrated under reduced pressure. Automated flash column chromatography (SiO_2_; 10 → 60% Et_2_O/pentane) afforded exclusively α-configured glycoside **15** (109 mg, 0.20 mmol, 67%) as a clear foam. **^1^H NMR** (400 MHz, CDCl_3_, HH-COSY, HSQC): δ 7.53 (d, *J* = 8.8 Hz, 1H, CH_arom_), 7.12 (d, *J* = 2.4 Hz, 1H, CH_arom_), 7.03 (dd, *J* = 8.8, 2.5 Hz, 1H, CH_arom_), 6.19 (q, *J* = 1.2 Hz, 1H, H-3’ MU), 5.71 (d, *J* = 3.4 Hz, 1H, H-1), 5.33 (dd, *J* = 10.9, 2.9 Hz, 1H, H-3), 4.79 (dd, *J* = 3.0, 1.0 Hz, 1H, H-4), 4.21 (dd, *J* = 12.9, 2.3 Hz, 1H, H-6), 4.12 – 4.03 (m, 2H, H-2, H-6), 3.85 – 3.81 (m, 1H, H-5), 2.41 (d, *J* = 1.2 Hz, 3H, CH_3_ MU), 2.21 (s, 3H, CH_3_ Ac), 1.07 (s, 9H, SiC(CH_3_)_3_), 1.02 (s, 9H, SiC(CH_3_)_3_); **^13^C NMR** (101 MHz, CDCl_3_, HSQC): δ 170.7 (C_q_), 161.0, 159.0, 155.1, 152.3, 125.9 (CH_arom_), 115.4 (C_q_), 113.7 (CH_arom_), 113.2 (C-3’ MU), 104.3 (CH_arom_), 97.4 (C-1), 71.2 (C-3), 70.0 (C-4), 68.4 (C-5), 66.7 (C-6), 56.7 (C-2), 27.7 (SiC(*C*H_3_)_3_), 27.3, 23.4 (Si*C*(CH_3_)_3_), 21.0 (CH_3_ Ac), 20.9 (Si*C*(CH_3_)_3_), 18.8 (CH_3_ MU); **ESI** **HRMS**: [M+Na^+^] calculated for: C_26_H_35_N_3_NaO_8_Si 568.2086; found 568.2087.

**4-Methylumbelliferyl-2-acetamido-2-deoxy-3-*O*-acetyl-4,6-*O*-(di-*tert*- butylsilylene)-α-D-galactopyranoside (16)**

Azide **15** (52 mg, 96 μmol, 1.0 equiv.) was dissolved in anhydrous THF (1 mL, 0.1 M), and PtO_2_ (6.6 mg, 28 μmol, 0.3 equiv.) was added. The reaction mixture was thoroughly purged with N_2_ and then placed under a H_2_ atmosphere. The reaction was stirred for 1.5 h, after which full conversion was observed (TLC: R*_f_* = 0.4, MeOH/DCM 2:18 v/v). The suspension was then purged with N_2_, filtered over Celite®, and rinsed with EtOAc. The filtrate was concentrated under reduced pressure and the crude intermediate was dissolved in anhydrous pyridine (1 mL, 0.1 M) and cooled on ice. Ac_2_O (14 μL, 144 μmol, 1.5 equiv.) was added and the mixture stirred overnight. Upon full conversion (TLC: R*_f_* = 0.4, EtOAc/pentane 6:4 v/v), the reaction was quenched with MeOH and diluted with EtOAc and sat. aq. NaHCO_3_. The aqueous layer was extracted with EtOAc (3 x) and the combined organic layers were washed with brine, dried over Na_2_SO_4_, filtered, and concentrated *in vacuo*. The crude product was purified by automated flash column chromatography (SiO_2_; 10 → 60% Et_2_O/pentane) to afford acetamide **16** (46 mg, 82 μmol, 85% over 2 steps) as a clear foam. **^1^H NMR** (400 MHz, CDCl_3_, HH-COSY, HSQC): δ 7.54 (d, *J* = 8.6 Hz, 1H, CH_arom_), 7.09 – 7.01 (m, 2H, CH_arom_), 6.18 (q, *J* = 1.2 Hz, 1H, H-3’ MU), 5.99 (d, *J* = 9.1 Hz, 1H, NH), 5.74 (d, *J* = 3.5 Hz, 1H, H-1), 5.23 (dd, *J* = 11.3, 2.9 Hz, 1H, H-3), 4.98 (ddd, *J* = 11.4, 9.2, 3.5 Hz, 1H, H-2), 4.64 (d, *J* = 3.0 Hz, 1H, H-4), 4.20 (dd, *J* = 12.8, 2.2 Hz, 1H, H-6), 4.08 (dd, *J* = 12.7, 1.7 Hz, 1H, H-6), 3.81 – 3.76 (m, 1H, H-5), 2.41 (d, *J* = 1.3 Hz, 3H, CH_3_ MU), 2.16 (s, 3H, CH_3_ Ac), 2.00 (s, 3H, CH_3_ Ac), 1.08 (d, *J* = 32.9 Hz, 18H, Si(C(CH_3_)_3_)_2_); **^13^C NMR** (101 MHz, CDCl_3_): δ 171.9 (C_q_), 170.4, 161.0, 159.1, 154.9, 152.4, 125.9 (CH_arom_), 115.2 (C_q_), 113.0 (C-3’ MU), 112.9 (CH_arom_), 104.7, 96.9 (C-1), 70.6 (C-3), 70.3 (C-4), 68.6 (C-5), 66.8 (C-6), 47.1 (C-2), 27.6 (SiC(*C*H_3_)_3_), 27.3, 23.4 (Si*C*(CH_3_)_3_), 23.4 (CH_3_ Ac), 21.1, 20.9 (Si*C*(CH_3_)_3_), 18.8 (CH_3_ MU); **ESI** **HRMS**: [M+Na^+^] calculated for: C_28_H_39_NNaO_9_Si 584.2286; found 584.2289.

**4-Methylumbelliferyl-2-acetamido-2-deoxy-4,6-*O*-(di-*tert*- butylsilylene)-α-D-galactopyranoside (17)**

Compound **16** (4.22 g, 0.75 mmol, 1.0 equiv.) was dissolved in anhydrous MeOH (7.5 mL, 0.1 M) and NaOMe (8.1 mg, 0.15 mmol, 0.2 equiv.) was added. The solution was stirred overnight, after which full conversion was observed (TLC: R*_f_* = 0.2, EtOAc/pentane 8:2 v/v). The reaction was quenched with Amberlite™ IR-120 resin (H^+^ form), until pH 5 was reached. The residual solvent was removed under reduced pressure, and the crude product was purified by automated flash column chromatography (SiO_2_; 1 → 10% MeOH/DCM) to afford alcohol **17** (334 mg, 0.64 mmol, 86%) as a clear foam. **^1^H NMR** (400 MHz, CDCl_3_, HH-COSY, HSQC): δ 7.53 (d, *J* = 8.5 Hz, 1H, CH_arom_), 7.05 – 7.00 (m, 2H, CH_arom_), 6.18 (q, *J* = 1.2 Hz, 1H, H-3’ MU), 5.78 (d, *J* = 8.8 Hz, 1H, NH), 5.73 (d, *J* = 3.4 Hz, 1H, H-1), 4.64 (ddd, *J* = 10.8, 8.8, 3.4 Hz, 1H, H-2), 4.51 (dd, *J* = 3.3, 1.2 Hz, 1H, H-4), 4.22 (dd, *J* = 12.6, 2.2 Hz, 1H, H-6), 4.09 (dd, *J* = 12.7, 1.6 Hz, 1H, H-6), 3.95 – 3.86 (m, 1H, H-3), 3.78 – 3.73 (m, 1H, H-5), 2.65 (d, *J* = 9.7 Hz, 1H, 3-OH), 2.41 (d, *J* = 1.2 Hz, 3H, CH_3_ MU), 2.06 (s, 3H, CH_3_ Ac), 1.11 (s, 9H, SiC(CH_3_)_3_), 1.06 (s, 9H, SiC(CH_3_)_3_); **^13^C NMR** (101 MHz, CDCl_3_, HSQC): δ 171.2 (C_q_), 161.0, 159.1 (C_q_), 155.0, 152.3, 125.9 (CH_arom_), 115.3 (C_q_), 113.2 (C-3’ MU), 112.9 (CH_arom_), 104.8, 97.1 (C-1), 72.6 (C-4), 69.5 (C-3), 68.7 (C-5), 66.8 (C-6), 49.9 (C-2), 27.7 (SiC(*C*H_3_)_3_), 27.4, 23.6 (CH_3_ Ac), 23.5 (Si*C*(CH_3_)_3_), 21.0 (Si*C*(CH_3_)_3_), 18.8 (CH_3_ MU); **ESI** **HRMS**: [M+Na^+^] calculated for: C_26_H_37_NNaO_8_Si 542.2181; found 542.2184.

**Sodium 4-Methylumbelliferyl-2-acetamido-2-deoxy-3-*O*-sulfo-4,6-*O*-(di-*tert*-butylsilylene)-α-D-galactopyranoside (18)**

Alcohol **17** (247 mg, 0.47 mmol, 1.0 equiv.) was dissolved in anhydrous DMF (5 mL, 0.1 M). SO_3_·pyridine (226 mg, 1.42 mmol, 3.0 equiv.) was added, and the mixture was stirred overnight. Upon full conversion (TLC: R*_f_* = 0.2, EtOAc/pentane 8:2 v/v), anhydrous pyridine (0.1 mL, 1.24 mmol, 2.6 equiv.) was added, and the mixture was stirred for another 30 min. Subsequently, the solvent was removed under reduced pressure, and the crude product was purified by automated flash column chromatography (SiO_2_, dry loading on Celite®; 1 → 20% MeOH/DCM) to afford sulfate **18** (272 mg, 0.42 mmol, 90%) as a white powder. **^1^H NMR** (400 MHz, MeOD, HH-COSY, HSQC): δ 7.71 (d, *J* = 8.8 Hz, 1H, CH_arom_), 7.15 (dd, *J* = 8.8, 2.4 Hz, 1H, CH_arom_), 7.07 (d, *J* = 2.4 Hz, 1H, CH_arom_), 6.19 (q, *J* = 1.3 Hz, 1H, H-3’ MU), 5.83 (d, *J* = 2.6 Hz, 1H, H-1), 4.99 (dd, *J* = 2.3, 0.9 Hz, 1H, H-4), 4.83 – 4.74 (m, 2H, H-2, H-3), 4.28 (dd, *J* = 12.7, 2.1 Hz, 1H, H-6), 4.03 (dd, *J* = 12.7, 1.7 Hz, 1H, H-6), 3.94 – 3.86 (m, 1H, H-5), 2.44 (d, *J* = 1.2 Hz, 3H, CH_3_ MU), 2.01 (s, 3H, CH_3_ Ac), 1.09 (d, *J* = 11.2 Hz, 18H, Si(C(CH_3_)_3_)_2_); **^13^C NMR** (101 MHz, MeOD, HSQC): δ 174.0 (C_q_), 163.2, 161.1 (C_q_), 155.9, 155.4, 127.5 (CH_arom_), 116.2 (C_q_), 114.9 (CH_arom_), 113.0 (C-3’ MU), 105.2 (CH_arom_), 98.2 (C-1), 75.3 (C-3), 72.4 (C-4), 70.2 (C-5), 68.0 (C-6), 49.0 (C-2), 28.2 (SiC(*C*H_3_)_3_), 28.0, 24.2 (Si*C*(CH_3_)_3_), 22.8 (CH_3_ Ac), 21.7 (Si*C*(CH_3_)_3_), 18.7 (CH_3_ MU); **ESI** **HRMS**: [M+H^+^] calculated for: C_26_H_38_NO_11_SSi 600.1929; found 600.1938.

**Sodium 4-methylumbelliferyl-2-acetamido-2-deoxy-3-*O*-sulfo-α-D-galactopyranoside (3)**

Sulfate **18** (37 mg, 60 μmol, 1.0 equiv.) was dissolved in anhydrous pyridine (1.2 mL, 0.05 M) and cooled on ice. HF-pyridine (70% w/w; 60 μL) was added and the mixture was stirred overnight at room temperature. Upon full conversion (TLC: R*_f_* = 0.3, MeOH/DCM 2:8 v/v), the reaction mixture was quenched with sat. aq. NaHCO_3_ and stirred for 1 h. Automated flash column chromatography (SiO_2_, dry loading on Celite®; 5 → 35% MeOH/DCM), followed by Na^+^-Dowex™ 50WX4 ion exchange and lyophilization afforded MU-α-3S-GalNAc **3** (24.8 mg, 52 μmol, 86%) as a white powder. **^1^H NMR** (500 MHz, MeOD, HH-COSY, HSQC): δ 7.70 (d, *J* = 8.8 Hz, 1H, CH_arom_), 7.17 (dd, *J* = 8.8, 2.5 Hz, 1H, CH_arom_), 7.14 (d, *J* = 2.3 Hz, 1H, CH_arom_), 6.19 (d, *J* = 1.3 Hz, 1H, H-3’ MU), 5.77 (d, *J* = 3.5 Hz, 1H, H-1), 4.75 (dd, *J* = 11.3, 2.9 Hz, 1H, H-3), 4.64 (dd, *J* = 11.4, 3.5 Hz, 1H, H-2), 4.41 (dd, *J* = 3.0, 1.2 Hz, 1H, H-4), 3.92 (app. td, *J* = 6.1, 1.3 Hz, 1H, H-5), 3.71 (d, *J* = 6.0 Hz, 2H, H-6), 2.44 (d, *J* = 1.2 Hz, 3H, CH_3_ MU), 2.00 (s, 3H, CH_3_ Ac); **^13^C NMR** (126 MHz, MeOD, HSQC): δ 174.0 (C_q_), 163.3, 161.3, 156.0, 155.4, 127.4 (CH_arom_), 116.2 (C_q_), 115.2 (CH_arom_), 113.0 (C-3’ MU), 105.3 (CH_arom_), 98.1 (C-1), 75.9 (C-3), 73.8 (C-5), 68.3 (C-4), 62.3 (C-6), 49.7 (C-2), 22.7 (CH_3_ Ac), 18.6 (CH_3_ MU); **ESI** **HRMS**: [M+H^+^] calculated for: C_18_H_22_NO_11_S 458.0763; found 458.0767.

# References

[1] P. R. Evans, G. N. Murshudov, *Acta Crystallogr D Biol Crystallogr* **2013**, *69*, 1204-1214.

[2] L. Potterton, J. Agirre, C. Ballard, K. Cowtan, E. Dodson, P. R. Evans, H. T. Jenkins, R. Keegan, E. Krissinel, K. Stevenson, A. Lebedev, S. J. McNicholas, R. A. Nicholls, M. Noble, N. S. Pannu, C. Roth, G. Sheldrick, P. Skubak, J. Turkenburg, V. Uski, F. von Delft, D. Waterman, K. Wilson, M. Winn, M. Wojdyr, *Acta Crystallogr D Struct Biol* **2018**, *74*, 68-84.

[3] A. J. McCoy, R. W. Grosse-Kunstleve, P. D. Adams, M. D. Winn, L. C. Storoni, R. J. Read, *J Appl Crystallogr* **2007**, *40*, 658-674.

[4] A. A. Vagin, R. A. Steiner, A. A. Lebedev, L. Potterton, S. McNicholas, F. Long, G. N. Murshudov, *Acta Crystallogr D Biol Crystallogr* **2004**, *60*, 2184-2195.

[5] K. Yamashita, M. Wojdyr, F. Long, R. A. Nicholls, G. N. Murshudov, *Acta Crystallogr D Struct Biol* **2023**, *79*, 368-373.

[6] P. Emsley, B. Lohkamp, W. G. Scott, K. Cowtan, *Acta Crystallogr D Biol Crystallogr* **2010**, *66*, 486-501.

[7] M. C. Courtinduchateau, A. Veyrieres, *Carbohyd Res* **1978**, *65*, 23-33.

[8] S. Park, I. Shin, *Org Lett* **2007**, *9*, 619-622.

[9] K. E. Osterlid, R. Cergano, H. S. Overkleeft, G. A. van der Marel, J. D. C. Codée, *Chem-Eur J* **2025**, *31*.

[10] B. Hagen, J. H. M. van Dijk, Q. J. Zhang, H. S. Overkleeft, G. A. van der Marel, J. D. C. Codée, *Org Lett* **2017**, *19*, 2514-2517.

# NMR spectra

^1^H NMR, 400 MHz, CDCl_3_ of **5**

^13^C NMR, 101 MHz, CDCl_3_ of **5**

HH-COSY NMR, CDCl_3_ of **5**

HSQC NMR, CDCl_3_ of **5**

^1^H NMR, 400 MHz, DMSO of **6**

^13^C NMR, 101 MHz, DMSO of **6**

HH-COSY NMR, DMSO of **6**

HSQC NMR, DMSO of **6**

^1^H NMR, 400 MHz, MeOD of **1**

^13^C NMR, 101 MHz, MeOD of **1**

HH-COSY NMR, MeOD of **1**

HSQC NMR, MeOD of **1**

^1^H NMR, 400 MHz, DMSO of **7** ^13^C NMR, 101 MHz, DMSO of **7**

HH-COSY NMR, DMSO of **7** HSQC NMR, DMSO of **7**

^1^H NMR, 400 MHz, MeOD of **8** ^13^C NMR, 101 MHz, MeOD of **8**

HH-COSY NMR, MeOD of **8** HSQC NMR, MeOD of **8**

^1^H NMR, 400 MHz, DMSO of **9**

^13^C NMR, 101 MHz, DMSO of **9**

HH-COSY NMR, DMSO of **9**

HSQC NMR, DMSO of **9**

^1^H NMR, 400 MHz, MeOD of **2**

^13^C NMR, 101 MHz, MeOD of **2**

HH-COSY NMR, MeOD of **2**

HSQC NMR, MeOD of **2**

^1^H NMR, 400 MHz, CDCl_3_ of **S1**

^13^C NMR, 101 MHz, CDCl_3_ of **S1**

HH-COSY NMR, CDCl_3_ of **S1**

HSQC NMR, CDCl_3_ of **S1**

^1^H NMR, 400 MHz, CDCl_3_ of **11**

^13^C NMR, 101 MHz, CDCl_3_ of **11**

HH-COSY NMR, CDCl_3_ of **11**

HSQC NMR, CDCl_3_ of **11**

^1^H NMR, 400 MHz, CDCl_3_ of **12**

^13^C NMR, 101 MHz, CDCl_3_ of **12**

HH-COSY NMR, CDCl_3_ of **12**

HSQC NMR, CDCl_3_ of **12**

^1^H NMR, 400 MHz, CDCl_3_ of **13**

^13^C NMR, 101 MHz, CDCl_3_ of **13**

HH-COSY NMR, CDCl_3_ of **13**

HSQC NMR, CDCl_3_ of **13**

^1^H NMR, 400 MHz, CDCl_3_ of **15**

^13^C NMR, 101 MHz, CDCl_3_ of **15**

HH-COSY NMR, CDCl_3_ of **15**

HSQC NMR, CDCl_3_ of **15**

^1^H NMR, 400 MHz, CDCl_3_ of **16**

^13^C NMR, 101 MHz, CDCl_3_ of **16**

HH-COSY NMR, CDCl_3_ of **16**

HSQC NMR, CDCl_3_ of **16**

^1^H NMR, 400 MHz, CDCl_3_ of **17**

^13^C NMR, 101 MHz, CDCl_3_ of **17**

HH-COSY NMR, CDCl_3_ of **17**

HSQC NMR, CDCl_3_ of **17**

^1^H NMR, 400 MHz, MeOD of **18**

^13^C NMR, 101 MHz, MeOD of **18**

HH-COSY NMR, MeOD of **18**

HSQC NMR, MeOD of **18**  ^1^H NMR, 400 MHz, MeOD of **3**

^13^C NMR, 101 MHz, MeOD of **3**

HH-COSY NMR, MeOD of **3**

HSQC NMR, MeOD of **3**
